# Tay Bridge Is a Negative Regulator of EGFR Signalling and Interacts with Erk and Mkp3 in the *Drosophila melanogaster* Wing

**DOI:** 10.1371/journal.pgen.1003982

**Published:** 2013-12-12

**Authors:** Cristina Molnar, Jose F. de Celis

**Affiliations:** Centro de Biología Molecular “Severo Ochoa,” CSIC and Universidad Autónoma de Madrid, Madrid, Spain; Harvard Medical School, Howard Hughes Medical Institute, United States of America

## Abstract

The regulation of Extracellular regulated kinase (Erk) activity is a key aspect of signalling by pathways activated by extracellular ligands acting through tyrosine kinase transmembrane receptors. In this process, participate proteins with kinase activity that phosphorylate and activate Erk, as well as different phosphatases that inactivate Erk by de-phosphorylation. The state of Erk phosphorylation affects not only its activity, but also its subcellular localization, defining the repertoire of Erk target proteins, and consequently, the cellular response to Erk. In this work, we characterise Tay bridge as a novel component of the EGFR/Erk signalling pathway. Tay bridge is a large nuclear protein with a domain of homology with human AUTS2, and was previously identified due to the neuronal phenotypes displayed by loss-of-function mutations. We show that Tay bridge antagonizes EGFR signalling in the *Drosophila melanogaster* wing disc and other tissues, and that the protein interacts with both Erk and Mkp3. We suggest that Tay bridge constitutes a novel element involved in the regulation of Erk activity, acting as a nuclear docking for Erk that retains this protein in an inactive form in the nucleus.

## Author Summary

Extracellular regulated kinases (Erk) mediate signalling by pathways activated by tyrosine kinase transmembrane receptors. The level of activated Erk depends on a highly regulated balance between cytoplasmic kinases and nuclear/cytoplasmic phosphatases, which determine the state of Erk phosphorylation. This affects Erk activity and its subcellular localization, defining the repertoire of Erk targets, and consequently, the cellular response to Erk. In this work, we use a genetic approach to characterise the gene *tay bridge* as a novel component of the EGFR/Erk signalling pathway. *Tay bridge* has a domain of homology with human AUTS2, and was previously identified due to the neuronal phenotypes displayed by loss-of-function mutations. We show that Tay bridge antagonizes EGFR signalling in the *Drosophila melanogaster* wing disc and other tissues, and that the protein interacts with both Erk and Mkp3. We suggest that Tay bridge constitutes a novel element involved in the regulation of Erk activity, acting as a nuclear docking for Erk that retains this protein in an inactive form in the nucleus. These results could provide important insights into the clinical consequences of AUTS2 mutations in humans, which are related to behavioural perturbations including autism, mental retardation, Attention Deficit Hyperactivity Disorder and alcohol drinking behaviour.

## Introduction

The Epidermal Growth Factor Receptor (EGFR) signalling pathway is a conserved module that plays multiple roles during development and tissue homeostasis in eukaryotic organisms [1]–[3]. The best-characterized functions of the pathway involve the EGFR downstream proteins Sos, Ras, Raf, Mek and Erk, the MAPK that is encoded by *rolled* in *Drosophila melanogaster* [4]. The activity of these core components is required in multiple developmental contexts, influencing cell proliferation, migration, apoptosis, epithelial integrity and cell fate acquisition [1], [5]. A key node in the regulation of EGFR signalling occurs at the level of Erk phosphorylation and de-phosphorylation by Mek and dual-specificity phosphatases, respectively [6]–[8]. In general, upon activation by Mek, the Erk serine/threonine kinase is transported into the nucleus, where it can phosphorylate specific transcription factors, regulating their activity and consequently gene expression. Erk is de-phosphorylated and inactivated by dual-specificity phosphatases, which promote Erk accumulation in an inactive state in the cytoplasm [2], [9].

The nucleus-cytoplasm compartmentalization of Erk is also regulated by several proteins acting as scaffolds, which influence the kinetics of Erk activation by favouring its association with upstream components, or that target Erk to different substrates by regulating its subcellular localization [10]–[11]. Thus, Kinase suppressor of Ras (Ksr) and MEK partner 1 (MP-1) facilitate the phosphorylation of Erk by Mek [11]–[16], whereas β-arrestin and Sef (Similar Expression to FGF genes) serve as scaffolds directing Erk activity toward different subcellular localizations and sets of target proteins [17]–[18]. In fact, because Erk lacks nuclear localization or export sequences, it appears that its subcellular compartmentalization is mostly determined by binding to scaffolds, anchors and substrates [8], [10], [19]. In the absence of active export, Erk tends to accumulate inside the nucleus, and it has been suggested that imported Erk binds to nuclear anchoring proteins that difficult its free diffusion to the cytoplasm [6].

The EGFR signalling system has been extensively characterised in *Drosophila*, an organism that has been instrumental to identify the intricacies of signalling regulation in vivo [1], [20]–[22]. Furthermore, the exquisite sensitivity of several developmental processes to variations in levels of EGFR signalling has driven the search and identification of many components of the pathway through genetic screens, expression profiling and cell culture experiments [22]–[25]. The wing disc, the epithelial tissue that gives rise to the adult wing and part of the thorax, is particularly sensitive to changes in the levels of EGFR signalling [26]–[27]. The function of EGFR in this tissue is required for cell proliferation and viability [28], for the specification of the wing disc and its territorial subdivision [26], [29]–[32], and also in cell fate choices affecting sensory organs and veins [33]–[34]. In this last process, the function of the pathway is needed to promote the formation of the veins, longitudinal stripes of cells that differentiate a cuticle thicker and more pigmented than the cuticle of inter-vein cells [35]–[36].

We conducted a gain-of-function screen aimed to identify genes regulating wing vein differentiation, expecting that some of these genes would encode novel components of the signalling pathways driving the formation of these structures [37]. In this screen, we identified a P-UAS insertion in the gene *tay bridge* (*tay*) that in combination with a vein-specific Gal4 driver causes the elimination of the longitudinal veins, a phenotype reminiscent of loss of EGFR activity in the developing veins [27], [37]. Tay encodes a large protein of 2486 amino acids expressed predominantly in the central nervous system [38]. Mutant *tay* flies present a constriction in the protocerebral bridge, and display reduced walking speed, reduced sensitivity to the effects of alcohol and defective compensation of rotatory stimuli during walking [38]–[39]. The Carboxi-terminal part of *Drosophila* Tay presents homology with mammalian AUTS2, a neuronal nuclear protein that is related to autism [40]–[41], mental retardation [42], [43], Attention Deficit Hyperactivity Disorder [44], and alcohol drinking behaviour [39]. *Auts2* expression is maximal in maturating neurons and declines as these cells become mature, suggesting that its function is required for neuronal differentiation [41], [45].

Here we report a genetic and developmental analysis of *tay* in the wing disc, and show that the function of Tay here is primarily related to the regulation of EGFR signalling. Thus, excess and loss of *tay* results in opposite phenotypes of loss- and extra veins, respectively, that are caused by changes in the levels of Erk activity. In addition, Tay level of expression modifies the phenotypic outcomes of altered EGFR signalling. We identify molecular interactions between Tay and Erk that might underline both the effects of Tay on Erk phosphorylation and the effects of Erk on Tay nuclear accumulation. All together, our results suggest that Tay is a novel component of the EGFR/Erk signalling pathway that regulates the nucleus/cytoplasm distribution of Erk.

## Results

### The phenotypes of *EP-866/Gal4* combinations are due to the over-expression of *tay*

**Figure 1:**
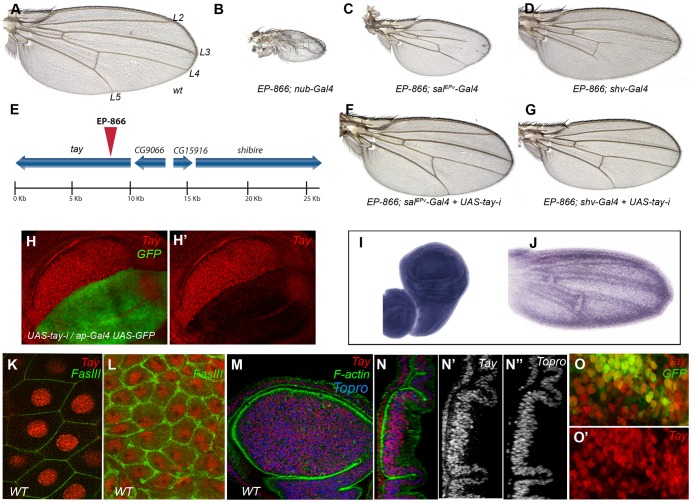
Genetic characterization of the *EP-866* insertion and expression analysis of *tay*. (A) Wild type wing (*wt*) indicating the position of the longitudinal veins L2–L5. (B–D) Combinations of *EP-866* with Gal4 drivers expressed in the entire wing blade and hinge (*EP-866/+; nub-Gal4/+*; B), in the central region of the wing blade (*EP-866; sal^EPv^-Gal4/+*; C) and in the pupal wing veins (*EP-866/+; shv-Gal4/+*; D). (E) Schematic representation of the genomic region around the insertion site of *EP-866*, showing the position and orientation of *tay*, *CG9066*, *CG15916* and *shibir*e coding regions. (F–G) Rescue of the *EP-866/Gal4* phenotype by expression of RNA interference directed against *tay* (*UAS-tay-i*). In both cases, *EP-866; sal^EPv^-Gal4/+; UAS-tay-i/+* (F) and *EP-866/+; shv-Gal4/UAS-tay-i* (G) the phenotype is rescued (compare F with C and G with D). (H–H′) Expression of Tay (red) and GFP (green) in *ap-Gal4 UAS-GFP/+; UAS-tay-i/+* third instar wing discs. The red channel (H′) shows the strong reduction in Tay protein levels. (I–J) *in situ* hybridization with a *tay* probe in a late third instar wing disc (I) and in a pupal wing 28 h after puparium formation (J). (K–L) High magnification pictures of a salivary gland (K) and the peripodial membrane of the wing disc (L) showing the accumulation of Tay (red channel) in the cell nucleus. The expression of FasIII (green) shows the contour of the cells. (M–N″) Expression of Tay (red), F-actin (green) and To-Pro (blue) in a third instar wing disc. (N–N″) Orthogonal section of the disc showed in M showing the individual channels for Tay (N′) and To-Pro (N″). (O–O′) Expression of Tay (red) in *EP-866; sal^EPv^-Gal4/+; UAS-GFP/+* discs, showing nuclear localization of Tay in cells over-expressing the protein. The expression of GFP is in green (O).

*EP-866* is a P-GS element inserted in the first intron of *tay*, and was selected in a gain-of-function screen designed to identify genes that, when over-expressed, affect the differentiation of the wing veins [37]. The combination of *EP-866* with a variety of Gal4 lines reduces the size of the wing and causes the partial loss of longitudinal veins (Fig. 1A–D; Fig. S1H–J). The most extreme phenotypes are observed in combinations of *EP-866* with Gal4 drivers expressed in the entire wing blade and hinge (*nub-Gal4/EP-866*; Fig. 1B). A weaker version of this phenotype is detected in combinations with a Gal4 driver expressed only in the central region of the wing blade (*sal^EPv^-Gal4/EP-866*; Fig. 1C). The reduction in wing size and loss of veins occurs in a compartment-specific manner, as they are also observed in combinations with the *hh-Gal4* and *ap-Gal4* drivers (Fig. S1J and data not shown). In all cases, the drastic reduction in wing size is associated with a reduction of cell proliferation, and not to the induction of cell death. Thus, wing discs of combinations between *EP-866* and Gal4 drivers show a very low number of mitotic cells and no activation of Caspase3 (Fig. S1A–G′). When the gene affected by the *EP-866* insertion is over-expressed during pupal development, the size of the wing is normal, but the veins fail to differentiate (Fig. 1D). *EP-866/Gal4* combinations also display phenotypes in other adult structures, including fusion of tarsal joints in the legs (*dll-Gal4*/*EP-866*; Fig. S1A, C), a significant reduction in the size of the eye (*ey-Gal4/EP-866*; data not shown) and loss of sensory organs in the thorax (*ap-Gal4/EP-866*; Fig. S1B, D). The strength of the *EP-866/Gal4* phenotype increases with the number of copies of both the Gal4 and the *EP-866* insertion (Fig. S1K–M).

The most likely candidate to cause the over-expression phenotype of *EP-866/Gal4* combinations is the gene *tay* (Fig. 1E). Nonetheless, the genes *CG15916* (5 Kb) and *shibire* (7 Kb) are close to the *EP-866* insertion, and adjacent to *tay* is located *CG9066*, which is oriented in the 3′ to 5′ direction of transcription regarding the UAS sequences of the P-GS insertion. We know that *tay*, *CG15916* and *shi* are over-expressed when *EP-866* is combined with the *sal^EPv^-Gal4* [37]. However, the phenotypes of wing size reduction and loss of veins observed in *EP-866/sal^EPv^-Gal4* and *EP-866/shv-Gal4* flies are suppressed when we introduced a *UAS-tay*-*RNAi* construct in these combinations (Fig. 1F–G, compare with 1C and D, respectively). In addition, the over-expression of Tay results in identical phenotypes of variable vein loss and wing size reduction (see below), indicating that *tay* causes the over-expression phenotypes of *EP-866/Gal4* combinations.

### Tay is a large nuclear protein related to human AUTS2

*tay* encodes a protein of 2486 amino-acids which most remarkable characteristic is a 30% of identity in the 1764–2019 amino acid region with a 486–782 stretch of the 1295 amino acid long human protein AUTS2 (Autism Susceptibility Candidate 2) (see below). The expression of *tay* occurs ubiquitously in all imaginal discs (Fig. 1I and data not shown), although we can also observe higher levels of expression in cells adjacent to the veins during pupal development (Fig. 1J). Tay is also expressed at other developmental stages, and during embryonic development its mRNA and protein are detected prominently in the central nervous system (Fig. S3E–G and data not shown). To visualize the accumulation of the Tay protein, we generated a specific polyclonal antibody (Fig. S2B), and found that the protein is present in the nucleus of all imaginal discs and salivary gland cells (Fig. 1K–N). The accumulation of Tay is very much reduced or lost in dorsal wing compartments expressing a *tay* RNA interference (Fig. 1H–H′). We also confirmed the specificity of this antibody by staining cells homozygous for a *tay* deficiency, where we found that the signal is completely lost (Fig. S2C–C′). The subcellular localization of the protein in wing discs over-expressing Tay is mostly nuclear, although some cytoplasmic staining is detected at higher level of over-expression (Fig. 1O–O′). These observations suggest that the adult phenotypes associated to Tay over-expression are caused by the accumulation of Tay at higher than normal levels in the nuclei of imaginal cells that normally express the gene. Interestingly, we also detected Tay in the cytoplasm of a subset of motoneurons in the central nervous system (CM and JFdC, data not shown), indicating that the protein subcellular localization is regulated in a cell-type specific manner.

### Loss of *tay* function causes excess of vein differentiation

**Figure 2:**
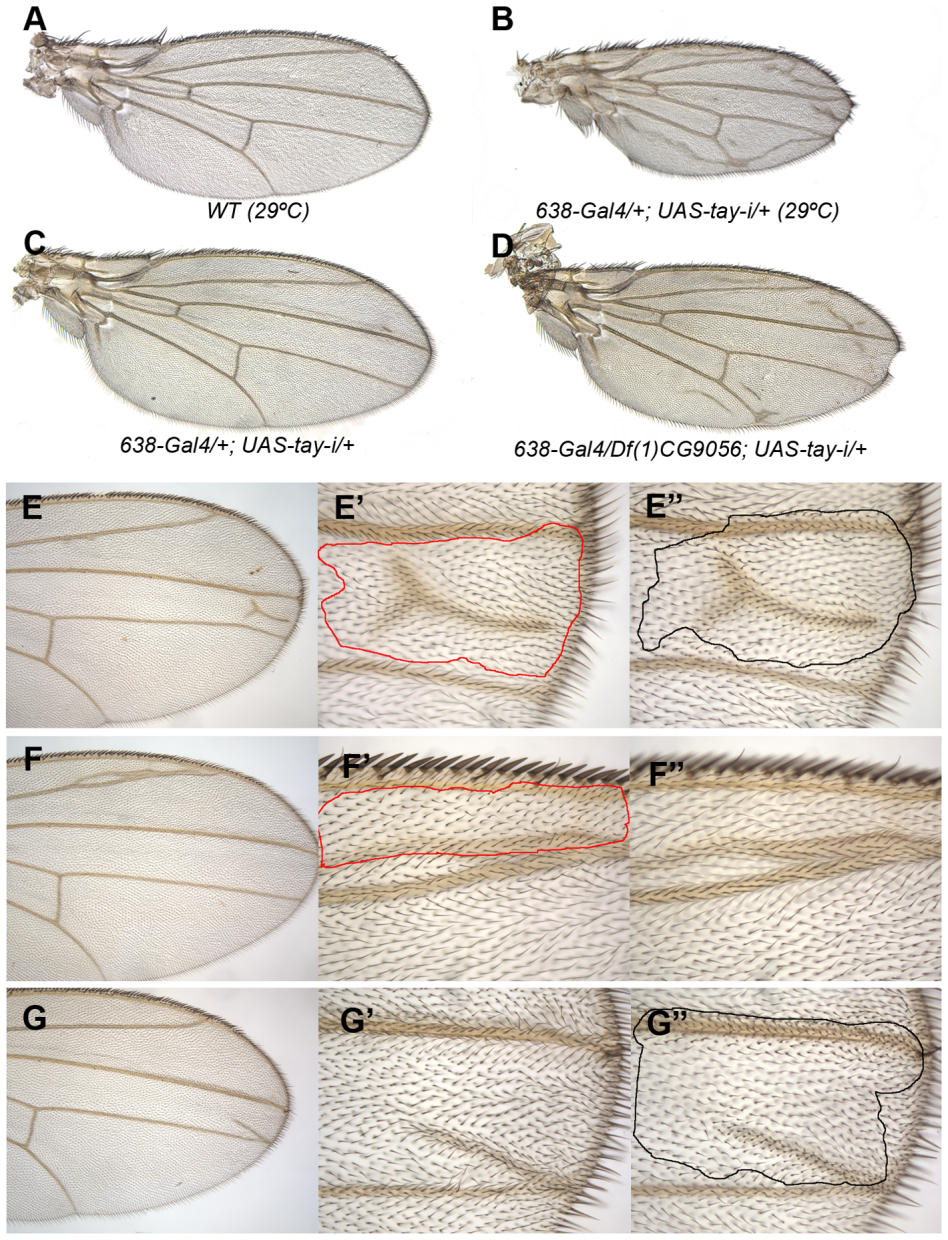
Loss-of-function analysis of *tay*. (A) Wild type wing grown at 29°C. (B) Wing size reduction, extra veins and loss of wing margin in *638-Gal4/+; UAS-tay-i/+* wings at 29°C. (C–D) The weak phenotype of *638-Gal4/+; UAS-tay-i/+* wings at 25°C (C) is increased in flies heterozygous for a *tay* deficiency (*638-Gal4/Df(1)tay; UAS-tay-i/+*; D). (E–G) Examples of mitotic recombination clones in flies of *f^36a^ Df(1)tay FRT18A/FRT18A UbiGFP; hsFLP/+* genotype. In E, example of a dorso-ventral clone located between the L3 and L4 veins (E′ and E″ are higher magnifications of the dorsal and ventral wing surfaces, respectively). In F example of a dorsal clone located anterior to the L2 vein. This clone (F′) differentiates vein cells and induces vein differentiation in the ventral wing surface (F″). In G example of a ventral clone located between the L3 and L4 veins that differentiate an ectopic vein in the ventral surface (G″) and induces vein differentiation in the dorsal surface (G′).

To identify the normal requirement of Tay during wing development, we reduced the levels of *tay* mRNA by expressing its RNA interference (*tay-RNAi*) in different domains of the wing disc. When *tay-RNAi* is expressed in the wing blade (*638-Gal4/UAS-tay-i*) the wings are reduced in size (32% smaller than wild type wings without changes in cellular size), display ectopic veins and show some defects in the most distal region of the wing margin (Fig. 2B). These phenotypes are caused by the reduction of *tay*, because they are enhanced in a genetic background with only one copy of the gene (Fig. 2C–D; *638-Gal4/+; Df(1)tay/UAS-tay-i*). To generate stronger loss-of-function conditions, we made two small deficiencies by transposition (*EP-866^Rev34^* and *EP-866^Rev40^*; see Fig. S2A), and a deficiency that eliminates *tay* and the adjacent gene *CG16952* (*Df(1)tay*; Fig. S2A). These alleles are embryonic lethal in homozygous flies, and consequently they were analysed in mitotic recombination clones. The results obtained in *Df(1)tay*, *EP-866^rev40^* and *EP-866^rev34^* clones were identical, with cells deficient for *tay* forming clones that differentiate ectopic veins in inter-vein territories (Fig. 2E–G and data not shown). Interestingly, only a fraction of the mutant cells in each clone differentiate as ectopic veins of normal thickness (Fig. 2E–G). These phenotypes were very similar to those observed in wings expressing the *tay-RNAi* (compare with Fig. 2A–D).

### Genetic interactions between Tay and upstream components of the EGFR pathway

**Figure 3:**
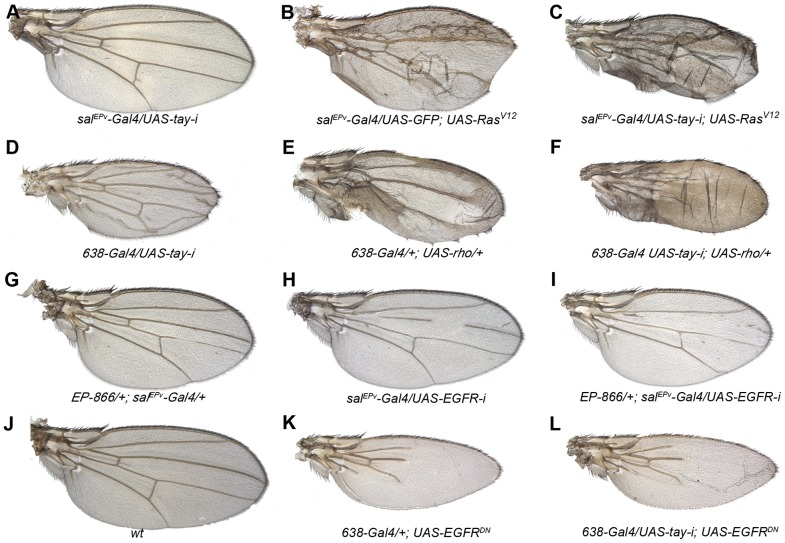
Genetic interactions between Tay and EGFR signalling. (A–C) The reduction of Tay (*sal^EPv^-Gal4/UAS-tay-i*; A) increases the phenotype of ectopic veins caused by Ras^V12^ over-expression (*sal^EPv^-Gal4/UAS-GFP; UAS-Ras^V12^/+*; B) in *sal^EPv^-Gal4/UAS-tay-i; UAS-Ras^V12^/+* (C).). (D–F) Interaction of *tay* and *rhomboid*. The reduction of Tay (*638-Gal4/UAS-tay-i*; D) and the increase in *rhomboid* expression (*638-Gal4/+; UAS-rho/+*; E) cause ectopic veins, and this phenotype is strongly enhanced in the double combination (*638-Gal4/UAS-tay-i; UAS-rho/+*; F). (G–I) Interaction of Tay and EGFR. The expression of Tay (*EP-866/+; sal^EPv^-Gal4/+*; G) enhances the phenotype of EGFR loss (*sal^EPv^-Gal4/UAS-EGFRi*; H) in *EP-866; sal^EPv^-Gal4/+; UAS- EGFRi/+* flies (I). Interaction of Tay and EGFR. The reduction of *tay* expression does not modify the strong loss of veins phenotype caused by the expression of a dominant negative form of EGFR (*638-Gal4/+; UAS-EGFR^DN^/+*; K) in *638-Gal4/UAS-tay-i; UAS-EGFR^DN^/+* flies (L). A wild type wing is shown in J.

The over-expression of *tay* in the wing imaginal disc prevents vein differentiation, macrochaetae formation and wing growth. Conversely, loss of *tay* function causes the formation of veins in inter-vein regions. These phenotypes are reminiscent to those caused by alterations in the levels of EGFR signalling, because loss of EGFR function impedes vein differentiation, and the increase in EGFR activity causes the formation of extra veins [27], [46]. To study the possible interactions between Tay and EGFR signalling, we made genetic combinations in which *tay* gain or loss of expression conditions were introduced in genetic backgrounds with modified EGFR activity. We find that the reduction of *tay* expression enhances the extra-vein phenotype caused by increased EGFR signalling. Thus, knock-down of *tay* enhances vein differentiation in Ras^V12^ (Fig. 3A–C) and ectopic *rhomboid* (Fig. 3D–F) backgrounds. These observations suggest that Tay function is necessary either to attenuate EGFR signalling or to reduce the response to particular levels of EGFR signalling. Compatible with these possibilities, Tay over-expression enhances the loss-of-vein phenotype caused by reduced activity of the pathway, for example in a situation when the expression of EGFR is reduced (Fig. 3G–I). Interestingly, the reduction of *tay* expression does not modify the complete loss of vein phenotype caused by strong reductions in EGFR signalling (Fig. 3J–L), indicating that Tay function is mostly required to modulate the levels of EGFR signalling once the pathway has been activated.

### Tay behaves as a negative regulator of the EGFR pathway

**Figure 4:**
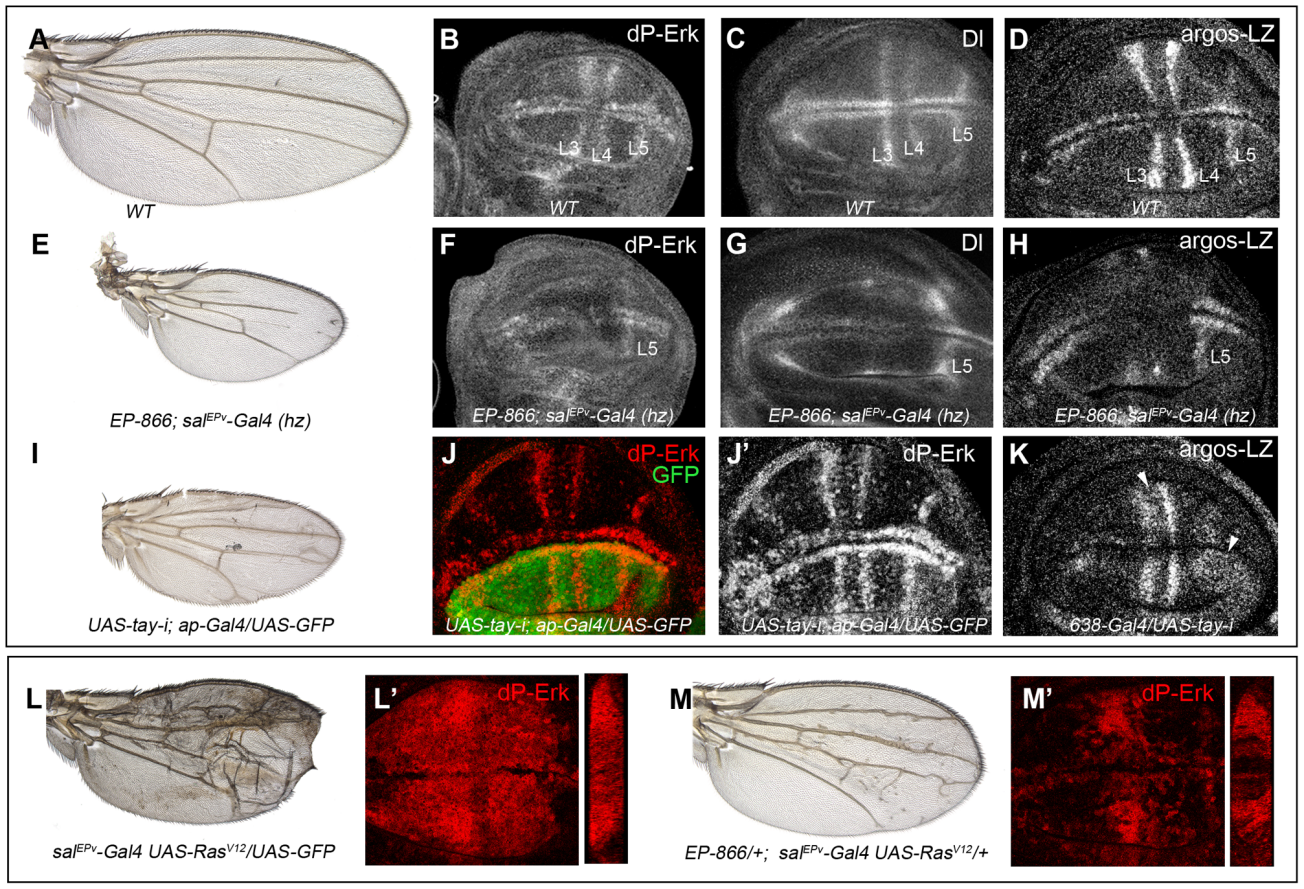
Effects of Tay on EGFR signalling. (A–D) Wild type wing (A) and late third instar wing discs showing the expression of dP-Erk (B), Delta (Dl, C) and *argos-lacZ* (D). (E–H) *EP-866; sal^EPv^-Gal4/sal^EPv^-Gal4* wing (E) showing reduced size and partial loss of the L2, L3 and L4 veins. Late third instar wing disc of the same genotype showing dP-Erk (F), Delta (G) and *argos-lacZ* (H) expression. Only the expression of dP-Erk, Delta and *argos-lacZ* in the L5 vein, which is outside the domain of *sal^EPv^-Gal4*, is present. (I–J′) Wing of *UAS-tay-i; ap-Gal4/UAS-GFP* genotype (I) and corresponding third instar wing disc showing the expression of dP-ERK (J–J′). Note the difference in expression levels between dorsal (labelled in green in J) and ventral inter vein cells. J′ corresponds to the red channel of J. (K) Expression of *argos-lacZ* in wing disc of *UAS-tay-i/638-Gal4* genotype. Note that the reduction of *tay* expression in the entire wing blade produces ectopic expression of *argos-lacZ*, mainly in the L3/L4 intervein and around the L5 vein (white arrowheads). (L–L′) Phenotype of Ras^V12^ over-expression (*sal^EPv^-Gal4 UAS-Ras^V12^/UAS-GFP*; L), and accumulation of dP-Erk (red; L′) in *sal^EPv^-Gal4 UAS-Ras^V12^/UAS-GFP* wing disc. (M–M′) The expression of Tay reduces the phenotype of *Ras^V12^* wings in *EP-866/+; sal^EPv^-Gal4/UAS-Ras^V12^* flies (M), and also reduces the accumulation of dP-Erk (red; M′) in *EP-866/+; sal^EPv^-Gal4/UAS-Ras^V12^* wing discs. Tangential sections through the length of the wing epithelium are shown to the right of L′ and M′.

To analyse whether changes in the expression of *tay* directly affect EGFR signalling, we monitored the levels of di-Phosphorylated Erk (dP-Erk) and the expression of the EGFR transcriptional targets *Delta* and *argos* in *tay* over-expression conditions. The accumulation of dP-Erk in wild type wing discs is maximal in the developing L3 and L4 longitudinal veins and in the marginal veins [34]; Fig. 4B). dP-Erk accumulation is strongly reduced in these territories when Tay is over-expressed in the wing blade (Fig. 4F, compare with 4B). The expression of *Delta* (*Dl*), which is regulated by EGFR signalling during imaginal development [47], is maximal in the veins L3, L4 and L5 and in the marginal veins in wild type wing discs (Fig. 4C). Over-expression of *tay* in the central region of the wing blade causes a reduction of *Dl* expression in the veins L3 and L4 (Fig. 4G, compare with 4C). The vein L5 is not affected, because it is located outside the domain of *sal^EPv^-Gal4* expression (Fig. 4F–G). Therefore, this vein serves as an internal control in these experiments. We also observed changes in the transcription of *argos*, which expression is also regulated by the EGFR pathway and is maximal in the veins L3, L4 and L5 and in the marginal veins in wild type wing discs [48]; Fig. 4D). Over-expression of *tay* reduces *argos-LacZ* expression (Fig. 4H, compare with 4D). In all cases, the changes in Erk phosphorylation and in *Dl*/*argos* gene expression caused by Tay over-expression were consistently stronger than the loss of vein phenotype observed in the corresponding adult wings, as these wings still differentiate some stretches of the L3 and L4 veins (Fig. 4E).

We also checked the effects of loss of Tay in the accumulation of dP-Erk. For this experiment we expressed *tay-RNAi* in the dorsal compartment of the wing (*ap-Gal4/UAS-tay-i*). In these discs the ventral compartment serves as an internal control. We observed that the reduction of *tay* expression increases dP-Erk accumulation in dorsal compartments compared with the ventral ones (Fig. 4J–J′). In addition, the expression of *tay-RNAi* in the entire wing blade (*638-Gal4/UAS-tay-i*) causes ectopic *argos-lacZ* expression (Fig. 4K, compare with 4D). Finally, we check whether excess of Tay can modulate dP-Erk accumulation under strong conditions of constitutive pathway activation. We find that Tay over-expression reduces the levels of dP-Erk induced by Ras^V12^ in the central region of the wing disc (Fig. 4L′, M′), and also the phenotype of ectopic veins caused by Ras^V12^ (Fig. 4L, M), suggesting that the negative effect of Tay on the activity of the EGFR pathway occurs downstream of Ras activation and affects the accumulation of dP-Erk. The effects of Tay loss and gain on dP-Erk accumulation were also detected in other imaginal discs, such as the eye disc (not shown) and the leg disc (Fig. S3C–D′), and also in embryos mutant for *tay* (Fig. S3A–B′), suggesting that Tay functions as a general modulator of Erk phosphorylation.

### Genetic interactions between Tay, Erk and Mkp3

**Figure 5:**
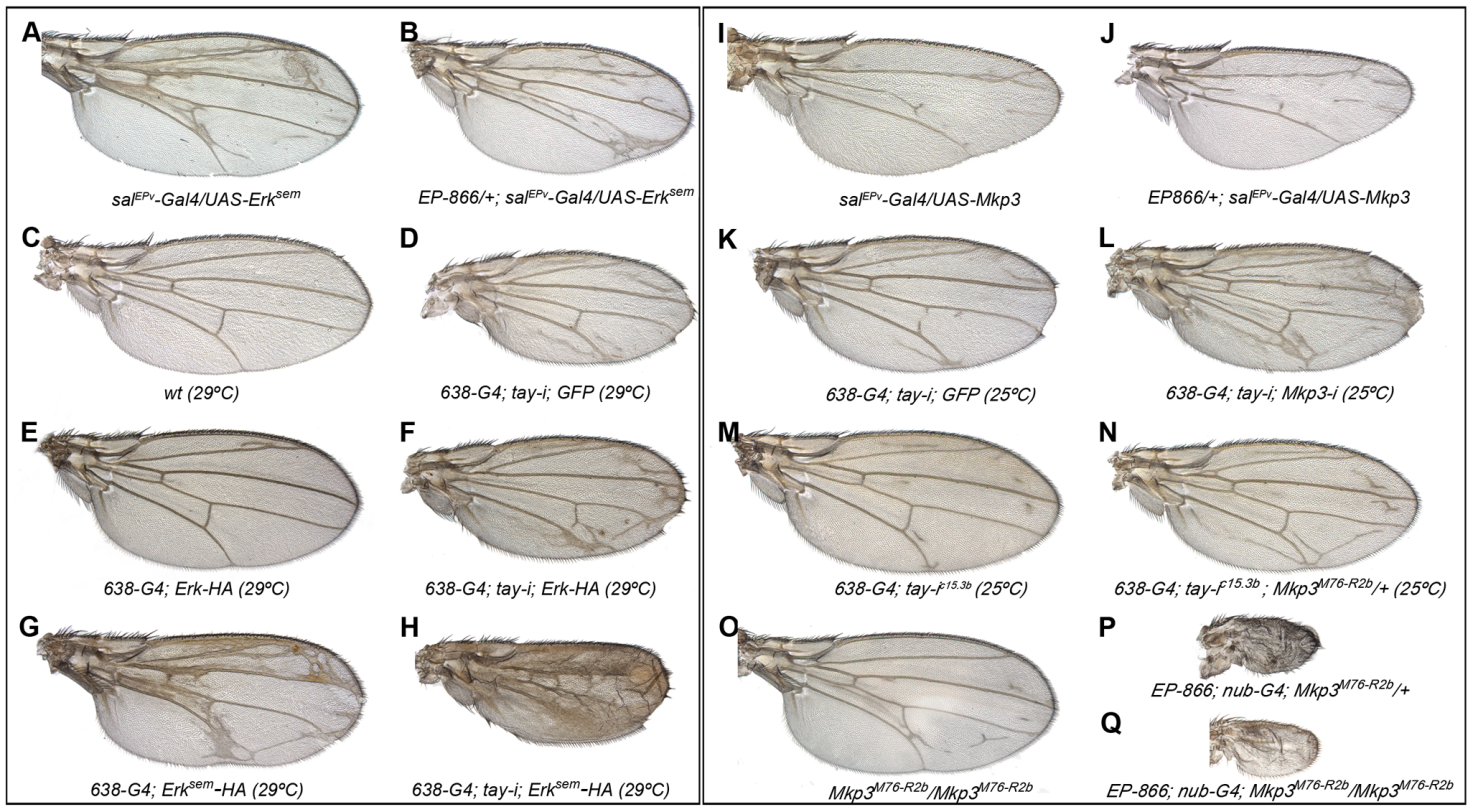
Genetic interactions between *tay*, *Mkp3* and *Erk*. (A–H) Genetic interactions between *tay* and *Erk*. (A–B) The over-expression of Tay does not supress the phenotype of ectopic veins caused by the expression of Erk^sem^ (*sal^PEv^-Gal4/UAS-Erk^sem^*; A) in *EP-866/+; sal^EPv^-Gal4/UAS-Erk^sem^* flies (B). (C–D) Control wild type wings grown at 29°C (C) and *638-Gal4/UAS-tay-i; UAS-GFP/+* wing showing ectopic veins in inter-vein regions and reduced size (D). (E–F) The ectopic vein phenotype of *638-Gal4/+; UAS-Erk-HA/+* flies (E) is enhanced when the expression of *tay* is reduced in *638-Gal4/UAS-tay-i; UAS-Erk-HA/+* flies (F). (G–H) The ectopic vein phenotype of *638-Gal4/+; UAS-Erk^sem^-HA/+* wings (G) is strongly increased when the expression of *tay* is reduced in *638-Gal4/UAS-tay-i; UAS-Erk^sem^-HA/+* flies (H). (I-P) Genetic interactions between *tay* and *Mkp3*. (I–J) The loss of veins phenotype of *sal^EPv^-Gal4/UAS-Mkp3* wings (I) is not modified when Tay is over-expressed in *EP-866*/+; *sal^EPv^-Gal4/UAS-Mkp3* wings (J). (K–L) The ectopic veins phenotype of *638-Gal4/UAS-tay-i; UAS-GFP/+* wings (K) is increased when the expression of *Mkp3* is also reduced in *638-Gal4/UAS-tay-i; UAS-Mkp3-i/+* flies (L). (M–N) The phenotype of ectopic veins of *638-Gal4/+; UAS-tay-i/+* wings (M) is increased in heterozygous *Mkp3* flies (*638-Gal4/UAS-tay-i; Mkp3^M76-R2b^/+*; N). (O–P) The phenotype of loss of veins and strong wing size reduction produced by the over-expression of Tay in the entire wing blade and hinge (*EP-866; nub-Gal4/+; Mkp3^M76-R2b^/+*; P) is not modified in homozygous *Mkp3* background (*EP-866; nub-Gal4/+; Mkp3^M76-R2b^/Mkp3^M76-R2b^*; Q). Control *Mkp3^M76-R2b^* homozygous wings are shown in panel O, and control heterozygous *Mkp3^M76-R2b^*/+ wings are indistinguishable from wild type.

The preferential nuclear localization of Tay and its effects on EGFR signalling and Erk phosphorylation prompted us to study the interactions between Tay and EGFR pathway components which subcellular localization shifts between the nucleus and the cytoplasm. We focussed this analysis on Erk and its specific phosphatase Mkp3. These proteins can interact with each other in the cytoplasm, where Mkp3 retains ERK and prevents its phosphorylation, and also in the nucleus, where Mkp3 de-phosphorylates and inactivates Erk [8], [49]. In addition, the phenotypes caused by the loss of Erk or Mkp3 are very similar to those cause by *tay* over-expression or loss of function, respectively. To study the genetic interactions between Tay and Erk we over-expressed wild type Erk or its mutant form *sevenmaker* (Erk^sem^), which bears a single amino acid substitution preventing Erk interactions with Mkp3 [50]–[51]. The use of Erk^sem^ allows the analysis of Erk over-expression conditions in the absence of its interaction with Mkp3. The formation of ectopic veins caused by a reduction in Tay levels is only weakly increased when the normal form of Erk is over-expressed (Fig. 5D–F). In contrast, loss of *tay* in a background of Erk^sem^ over-expression causes a strong increase in the differentiation of extra-vein tissue (Fig. 5H), compared with loss of only *tay* (Fig. 5D) or with Erk^sem^ over-expression (Fig. 5G). Interestingly, Tay over-expression reduces, but does not suppress, the ectopic veins caused by Erk*^sem^* (Fig. 5A–B). These results suggest that Erk^sem^ is much more effective when Tay levels are reduced, and, conversely, that Tay is less effective antagonizing Erk when this protein cannot interact with Mkp3.

In the case of Mkp3, the loss of veins caused by its over-expression (Fig. 5I) is not modified by loss (not shown) or excess of *tay* (Fig. 5J), confirming that Tay levels are not relevant upon a strong loss of Erk activation. In contrast, the formation of extra veins observed in *tay* loss-of-function conditions (Fig. 5K, M) depends on the gene dosage of *Mkp3*, becoming stronger in *Mkp3^M76-R2b^* heterozygous flies (Fig. 5N, compare with M) or upon expression of *Mkp3-RNAi* (Fig. 5L, compare with K). One possible explanation for these interactions is that Tay participates in the regulation of Erk inactivation, perhaps by promoting its de-phosphorylation. This possibility is compatible with the strong reduction of Erk phosphorylation caused by Tay over-expression, and implies that Tay over-expression phenotypes should be dependent on the presence and activity of Erk phosphatases such as Mkp3. However, we notice that the phenotype of Tay over-expression is not modified in *Mkp3* null mutant backgrounds (Fig. 5O–Q). Thus, although we cannot exclude a role of Mkp3 in Tay function, this result indicates that the effects of Tay over-expression are not mediated exclusively by the activity of *Mkp3*.

### Effects of Tay in the activation of ERK

**Figure 6:**
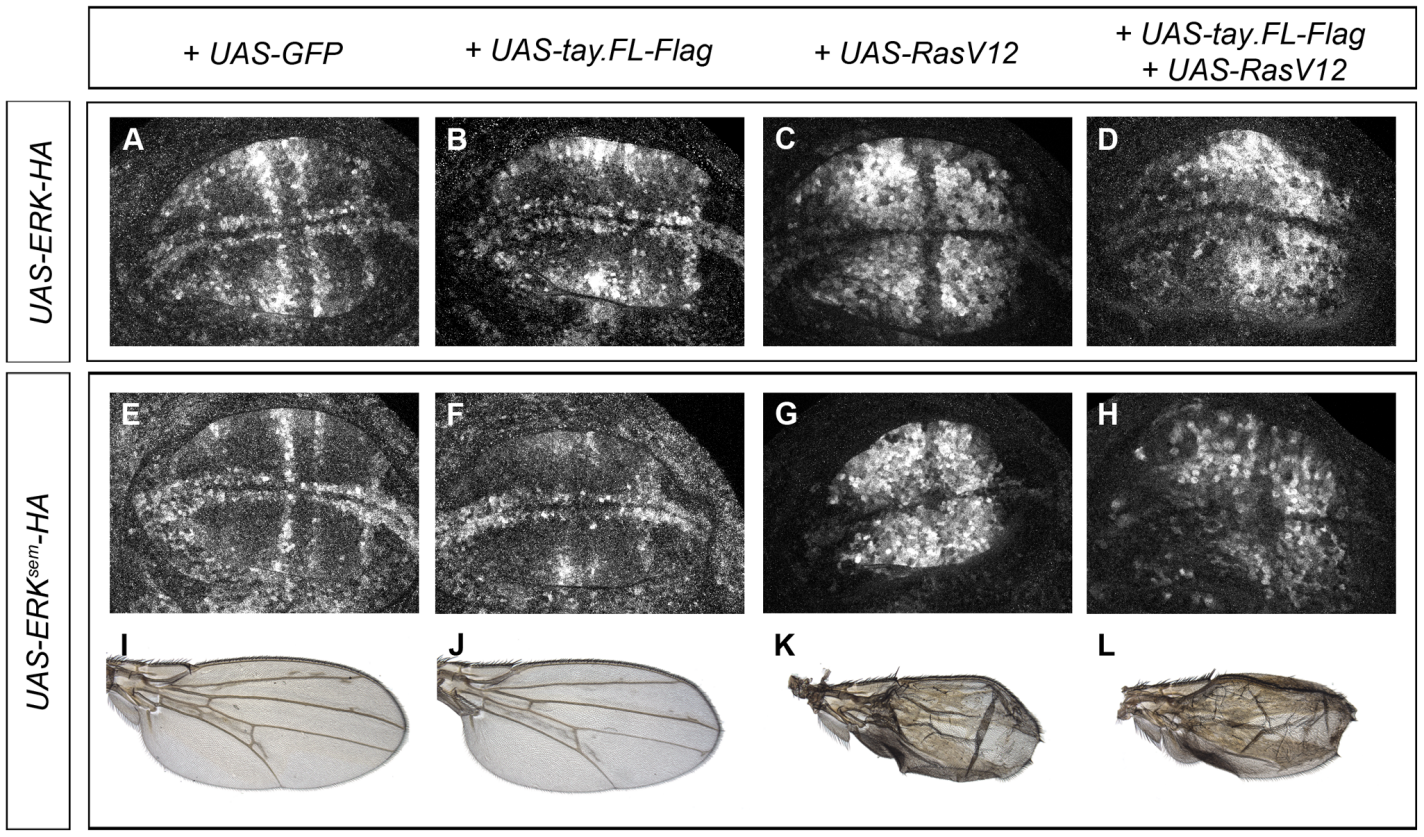
Effects of Tay over-expression in the activation of Erk. (A–D) Accumulation of dP-Erk in late third instar wing discs over-expressing Erk-HA. (A) Control *sal^EPv^-Gal4/UAS-Erk-HA*; *UAS-GFP/+* wing disc. (B) *sal^EPv^-Gal4/UAS-Erk-HA*; *UAS-tay.FL-Flag/+* wing disc. (C) *sal^EPv^-Gal4/UAS-Erk-HA*; *UAS-Ras^V12^/+* wing disc. (D) *sal^EPv^-Gal4/UAS-Erk-HA*; *UAS-Ras^V12^/UAS-tay.FL-Flag* wing disc. (E–F) Accumulation of dP-Erk in late third instar wing discs over-expressing Erk^sem^-HA. (E) Control *sal^EPv^-Gal4/UAS-Erk^sem^-HA*; *UAS-GFP/+* wing disc. (F) *sal^EPv^-Gal4/UAS-Erk^sem^-HA*; *UAS-tay.FL-Flag/+* wing disc. (G) *sal^EPv^-Gal4/UAS-Erk^sem^-HA*; *UAS-Ras^V12^/+* wing disc. (H) *sal^EPv^-Gal4/UAS-Erk^sem^-HA*; *UAS-Ras^V12^/UAS-tay.FL-Flag* wing disc. (I–L) Wings of *sal^EPv^-Gal4/UAS-Erk^sem^-HA*; *UAS-GFP/+* (I), *sal^EPv^-Gal4/UAS-Erk^sem^-HA*; *UAS-tay.FL-Flag/+* (J), *sal^EPv^-Gal4/UAS-Erk^sem^-HA*; *UAS-Ras^V12^/+* (K) and *sal^EPv^-Gal4/UAS-Erk^sem^-HA*; *UAS-Ras^V12^/UAS-tay.FL-Flag* (L) genotypes.

Next, we wanted to visualize the activation of Erk in genetic backgrounds where the level of Erk and Tay expression is changed and the activity of the EGFR pathway is increased. To this end, we made tagged forms of Tay (Tay-Flag), Erk (Erk-HA) and Erk^sem^ (Erk^sem^-HA) and studied the accumulation of dP-Erk in wing discs of different genotypes. The expression of Erk-HA and Erk^sem^-HA causes very weak (Erk-HA; Fig. 5E) or moderate (Erk^sem^-HA; Fig. 5G and 6I) extra veins. In none of these over-expression backgrounds we were able to detect changes in the pattern or level of dP-Erk accumulation (Fig. 6A and 6E). The reduction of Erk phosphorylation caused by Tay over-expression (Fig. 4) is still observed when either Erk-HA (Fig. 6B) or Erk^sem^-HA (Fig. 6F) is expressed in combination with Tay. The strong activation of the pathway caused by Ras^V12^ is also observed in backgrounds of Erk-HA or Erk^sem^-HA expression (Fig. 6C and G, respectively). The introduction of Tay in these backgrounds causes a moderate reduction in dP-Erk accumulation (Fig. 6D and H, compare with 6C and G), although the resulting phenotype of ectopic vein differentiation is not reduced (Fig. 6K–L). From these observations we conclude that Tay is still effective in promoting the de-phosphorylation of Erk under conditions of Erk and Erk^sem^ over-expression, but less so in backgrounds of strong pathway activation.

### Effects of Tay in the subcellular localization of Erk, Erk^sem^ and Mkp3

**Figure 7:**
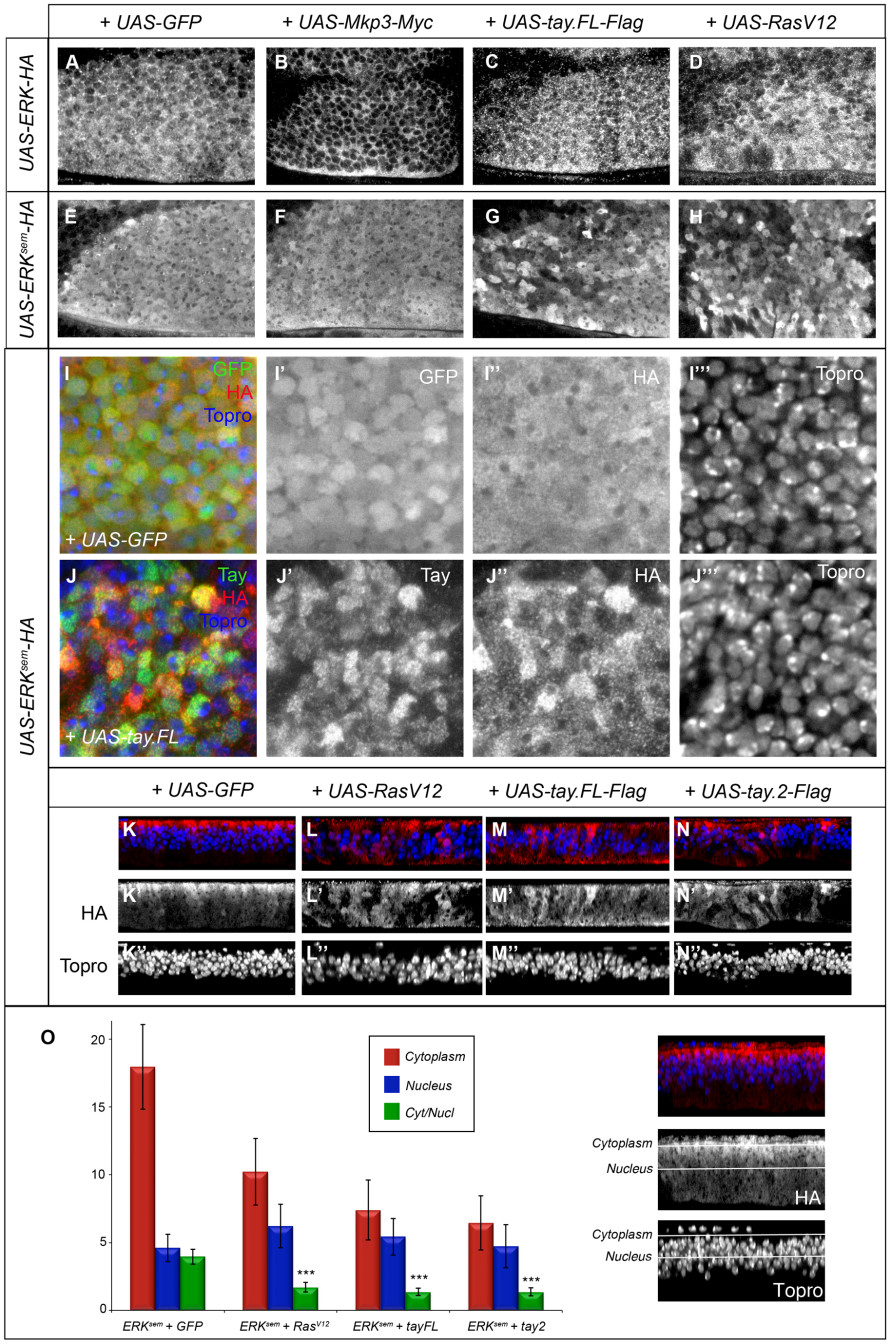
Subcellular localization of Erk in over-expression conditions. (A–B′) Subcellular localization of Erk (HA) in the anterior-dorsal region of wing discs of the following genotypes: *sal^EPv^-Gal4/UAS-Erk-HA*; *UAS-GFP/+* (A); *sal^EPv^-Gal4/UAS-Erk-HA; UAS-Mkp3-Myc/+* (B); *sal^EPv^-Gal4/UAS-Erk-HA*; *UAS-tay.FL-Flag/+* (C) and *sal^EPv^-Gal4/UAS-Erk-HA*; *UAS-Ras^V12^/+* (D). Note the cytoplasmic retention of Erk-HA in the presence of Mkp3-Myc (B) compared to the control (A). (E–H) Subcellular localization of Erk^sem^ (HA) in wing imaginal discs of the following genotypes: *sal^EPv^-Gal4/UAS-Erk^sem^-HA*; *UAS-GFP/+* (E); *sal^EPv^-Gal4/+; UAS-Erk^sem^-HA/UAS-Mkp3-Myc* (F); *sal^EPv^-Gal4/UAS-Erk^sem^-HA*; *UAS-tay.FL-Flag/+* (G) and *sal^EPv^-Gal4/UAS-Erk^sem^-HA*; *UAS-Ras^V12^/+* (D). Note the similar appearance of Erk^sem^ accumulation in the presence of Tay.FL-Flag (G) and Ras^v12^ (H). (I–J) Higher magnification pictures of wing discs showing the expression of Erk^sem^-HA (I″ and J″), Tay (J′) and To-pro (I′″ and J′″). The composite images are shown in panels I and J. In all cases Erk^sem^-HA was over-expressed in the central region of the wing disc in larvae of the *sal^EPv^-Gal4/UAS-Erk^sem^-HA*; *UAS-GFP/+* (I–I′″) and *sal^EPv^-Gal4/UAS-Erk^sem^-HA*; *UAS-tay.FL-Flag/+* genotype (J–J′″). Note the more heterogeneous appearance of Erk^sem^-HA in presence of Tay.FL (J–J′″) compare to controls (I–I′″). (K–N) Transverse sections through the wing disc showing the expression of Erk^sem^-HA (red) and To-pro (blue) in wing discs of *sal^EPv^-Gal4/UAS-Erk^sem^-HA*; *UAS-GFP/+* (K–K″), *sal^EPv^-Gal4/UAS-Erk^sem^-HA*; *UAS-ras^V12^/+* (L–L″), *sal^EPv^-Gal4/UAS-Erk^sem^-HA*; *UAS-tay.FL-Flag/+* (M-M″) and *sal^EPv^-Gal4/UAS-Erk^sem^-HA*; *UAS-tay.2-Flag/+* (N–N″). (O) Average values and standard deviation of Erk^sem^ signal intensity in 6 sections of 6 discs in each genotype (n = 36) taken from the apical (cytoplasm; red columns and see white line in the picture to the right) or medial (nucleus; blue columns and see white line in the picture to the right) level of the epithelium. The Cytoplasm/Nucleus ratio (green columns) was calculated as the average ratio of 36 pair of measures. In the three genotypes analysed the Cytoplasm/Nucleus ratio is significantly lower that control (p>0.005, t-student).

The subcellular localization of Mkp3 and Erk is dynamic, shifting between the nucleus and the cytoplasm [8], [49]. We wanted to analyse whether Tay influences the accumulation of these proteins in wing imaginal cells in over-expression conditions. First, we confirmed that Mkp3-Myc is preferentially localised in the cytoplasm (Fig. S4A–A′″), and that both Erk-HA and Erk^sem^-HA are detected in the nucleus and in the cytoplasm, with Erk-HA distributed at higher levels in the cytoplasm (Fig. 7A and E, respectively and Fig. S4C–C″ and E–E′″). The co-expression of Mkp3-Myc and Tay-Flag does not modify the preferential cytoplasmic (Mkp3) or nuclear (Tay) accumulation of these proteins (Fig. S4B–B′″). The co-expression of Mkp3-Myc and Erk-HA results in a clear cytoplasmic retention of Erk-HA (Fig. 7B, compare with A). In contrast, Mkp3-Myc does not modify the homogeneous nucleus-cytoplasm distribution of Erk^sem^-HA (Fig. 7F, compare with E). Neither the localization of Tay-Flag or Erk-HA changes when both are co-expressed in the same cells of the central region of the wing disc (Fig. 7C and Fig. S4D–D′″). In addition, the expression of Ras^V12^ does not affect the localization of Erk-HA, which is still localised in the nucleus and cytoplasm (Fig. 7D, compare with A, and Fig. S5A–A″).

In contrast, both Erk^sem^-HA and Tay-Flag display a heterogeneous distribution when co-expressed (Fig. 7G, J–J″ and Fig. S4F–F′″). We took higher magnification pictures of sections taken from the most anterior region of the *sal^EPv^-Gal4* domain of expression, because in these cells the level of over-expression are lower and Tay retains its nuclear localization (Fig. S7). We observed that the nuclear level of Erk^sem^-HA and Tay in each cell are not correlated (r^2^ = 0.09; n = 60). A similar heterogeneous distribution of ERK^sem^ was observed in a Ras^V12^ background (Fig. 7H and Fig. S5C–C″), and also when both Tay-Flag and Erk^sem^-HA were co-expressed in a Ras^v12^ background (Fig. S5D–D″). We do not understand the molecular bases for these changes in Erk^sem^ and Tay accumulation in the presence of each other or upon strong pathway activation, but they might be related to a dynamic regulation of protein turnover when Tay and Erk are co-expressed at higher levels. To get a quantitative view of Erk^sem^ nuclear-cytoplasmic localization, we took serial sections of the wing disc, quantified the levels of Erk^sem^ in the cytoplasm (apical in the epithelium; Fig. 7O) and nucleus (medial in the epithelium; Fig. 7O), and calculated the average cytoplasm/nucleus ratio of Erk^sem^ signal in different genetic backgrounds (Fig. 7K–O). These measures show that Erk^sem^ is mostly localised apically in the cell (cytoplasm), and that both the presence of Ras^v12^ (Fig. 7K) or Tay-Flag (Fig. 7L) strongly reduce the amount of cytoplasmic Erk^sem^ and weakly increase the level of nuclear Erk^sem^ (Fig. 7N). In this manner, the expression of either Ras^V12^ or Tay changes Erk^sem^ localization in a similar manner, but although both Tay and Ras^V12^ reduce the cytoplasm/nucleus ratio of Erk^sem^ accumulation, Erk activation, as visualised by the presence of dP-Erk (see Fig. 6), only occurs in Ras^V12^ conditions.

### Tay binds both ERK and MKP3

**Figure 8:**
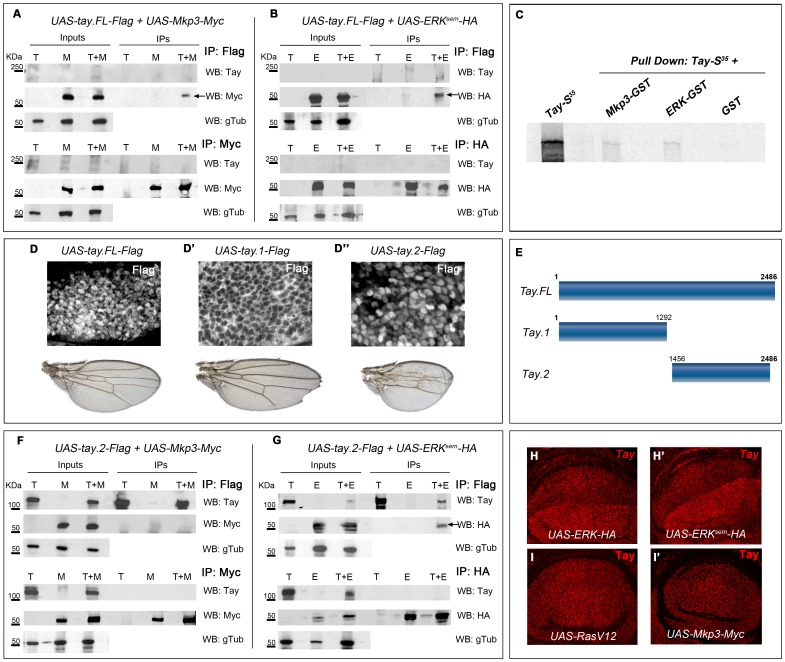
Tay interacts with Erk and Mkp3. (A–B) Interactions between Tay and Mkp3 (A) and between Tay and Erk (B). Embryo extracts were immunoprecipitated with anti-Flag (upper lines in A and B), anti-Myc (lower lines in A) and anti-HA (lower lines in B). Immunoprecipitates (IPs) and lysates (Inputs) were immunoblotted with anti-Myc (detecting Mkp3-Myc protein), anti-HA (detecting Erk^sem^-HA protein) and anti-Tay (we could not detect neither endogenous Tay nor Tay-Flag protein). In control IPs carried out with *da-Gal4/UAS-tay.FL-Flag* (T) and *da-Gal4/UAS-Mkp3-Myc* (M) embryos in A, and *da-Gal4/UAS-tay.FL-Flag* (T) and *da-Gal4/UAS-Erk^sem^-HA* (E) embryos in B, we did not detect any Tay, Mkp3 or Erk proteins. In Co-IPs experiments carried out with *da-Gal4/UAS-tay.FL-Flag; UAS-Mkp3-Myc/+* embryos (T+M) in A and *da-Gal4/UAS-tay.FL-Flag; UAS-Erk^sem^-HA/+* (T+E) embryos in B, we detected Mkp3-Myc protein in A and Erk^sem^-HA protein in B when the extracts were immunoprecipitated with anti-Flag (black arrows). (C) Pull down assay showing the interactions Tay-Mkp3 and Tay-Erk. Tay protein was in vitro translated and radiolabelled with Met^S35^. Tay was detected when it was incubated with Mkp3-GST protein and Erk-GST protein, but was not detected in control pulldowns with GST. (D–D″) Subcellular localization of Tay (Flag, white in D–D″) in wing imaginal discs of *sal^EPv^-Gal4/UAS-tay.FL-Flag* (D), *sal^EPv^-Gal4/UAS-tay.1-Flag* (D′) and *sal^EPv^-Gal4/UAS-tay.2-Flag* (D″) and their corresponding wings. (E) Schematic representation of Tay (Tay.FL) and the N-terminal (Tay.1) and C-terminal (Tay.2) forms. (F–G) Interactions between Tay.2 and Mkp3 (F) and Tay.2 and Erk (G). Embryo extracts were immunoprecipitated with anti-Flag (upper lines in F and G), anti-Myc (lower lines in F) and anti-HA (lower lines in G). Immunoprecipitates (IPs) and lysates (Inputs) were immunoblotted with anti-Myc (detecting Mkp3-Myc protein), anti-HA (detecting Erk^sem^-HA protein) and anti-Tay (detecting Tay.2-Flag protein). In control IPs carried out with *da-Gal4/UAS-tay.2-Flag* (T) and *da-Gal4/UAS-Mkp3-Myc* (M) embryos in F, and with *da-Gal4/UAS-tay.2-Flag* (T) and *da-Gal4/UAS-Erk^sem^-HA* (E) embryos in G, we did not detect any Tay.2, Mkp3 or Erk protein. In Co-IPs experiments carried out with *da-Gal4/UAS-tay.2-Flag; UAS-Mkp3-Myc/+* embryos (T+M) in F, we did not detect Mkp3-Myc protein when the extracts were immunoprecipitated with anti-Flag. In Co-IPs experiments carried out with *da-Gal4/UAS-tay.2-Flag; UAS-Erk^sem^-HA/+* embryos (T+E) in G, we detected Erk^sem^-HA protein when the extracts were immunoprecipitated with anti-Flag (black arrow). (H–I′) Accumulation of Tay in wing discs over-expressing Erk, Erk^sem^, Ras^V12^ and Mkp3. The over-expression is limited to the dorsal compartment of *ap-Gal4/UAS-Erk-HA* (H) and *ap-Gal4/UAS-Erk^sem^-HA* (H′) discs, or to the central domain of the wing blade in *sal^EPv^-Gal4/UAS-Ras^V12^* (I) and *sal^EPv^-Gal4/UAS-Mkp3-Myc* (I′) discs. Note the increased levels of Tay in dorsal compartments over-expressing Erk-HA and Erk^sem^-HA (H–H′).

We next considered the possibility that Tay might be directly interacting with Erk or Mkp3 in co-immunoprecipitation and pull-down experiments. Co-immunoprecipitation experiments were carried out from protein extracts obtained from embryos expressing combinations of Tay.FL-Flag, Mkp3-Myc, Erk^sem^-HA and Erk-HA (see Fig. S2D–E). Tay.FL-Flag was never detected in western blots, perhaps because the size of the protein prevents its transference to the membrane. However, when Tay-Flag is co-expressed with Mkp3-Myc or Erk^sem^-HA, we detected co-immunoprecipitation when the IP was made using anti-Flag and the western blot revealed using anti-Myc (Fig. 8A, line T+M from IP lanes) or anti-HA (Fig. 8B, line T+E from IP lanes). In protein extracts from embryos expressing only Mkp3-Myc or Erk^sem^-HA and IP with anti-Flag, we never detected Myc or HA (Fig. 8A–B, lines M and E, from IP lanes, respectively). The interaction between Tay and Erk and between Tay and Mkp3 might be direct, because they were also observed in pull-down experiments using in vitro translated Tay incubated with Erk-GST and Mkp3-GST fusion proteins (Fig. 8C).

To identify the region of Tay involved in these interactions, we made several truncated forms of the protein (Fig. 8E and data not shown), and expressed them in the wing disc. We found that the 1292 amino acid N-terminal fragment of Tay (Tay.1) is located exclusively in the cytoplasm (Fig. 8D′), and its over-expression does not affect the differentiation of veins (Fig. 8D′). In contrast, the 1030 amino acid C-terminal fragment (Tay.2) is accumulated preferentially in the nucleus (Fig. 8D″), similar to the full-length Tay-Flag protein (Fig. 8D). Interestingly, the expression of Tay.2 consistently results in stronger phenotypes of vein loss and reduced wing size than those caused by the over-expression of the full-length protein (Fig. 8D″ compare with D). This C-terminal fragment includes the domain of homology detected between Tay and human AUTS2. The distribution of Erk^sem^ is not modified in the presence of the N-terminal portion of Tay (data not shown). In contrast, Tay.2 results in the same changes in the cytoplasm/nucleus ratio of Erk^sem^ accumulation as Tay.FL (Fig. 7N–O).

The C-terminal 1030 amino acid Tay fragment (Tay.2) contains all the information necessary to regulate the subcellular localization of the protein, and also all the domains necessary to reproduce the effects of the full-length protein (see above). We repeated the immunoprecipitation experiments using this fragment, and found that Tay.2 retains its interaction with Erk^sem^ (Fig. 8G, line T+E, IP lanes), but loses its ability to interact with Mkp3 (Fig. 8F, line T+M, IP lanes). The failure of Tay.2 to interact with Mkp3 might increase the titration of ERK by Tay.2, explaining why Tay.2 interferes with EGFR signalling more efficiently than the full-length protein.

We also found that the levels of Tay accumulation in the nucleus are much higher than normal in cells over-expressing Erk or Erk^sem^ (Fig. 8H–H′ and Fig. S7A–D). As Erk or Erk^sem^ over-expression do not change the expression of *tay* (not show), these observations indicate that Erk increases the stability of Tay in the nucleus. This effect is independent of EGFR signalling, as neither Ras^V12^ nor Mkp3 over-expression modified the accumulation of endogenous (Fig. 8I–I′) or over-expressed Tay (Fig. S6A–B″). We conclude from these data that Tay can interact with Erk in the nucleus and that Erk protects Tay from degradation.

### The expression of human AUTS2 in the wing disc causes the formation of ectopic veins

**Figure 9:**
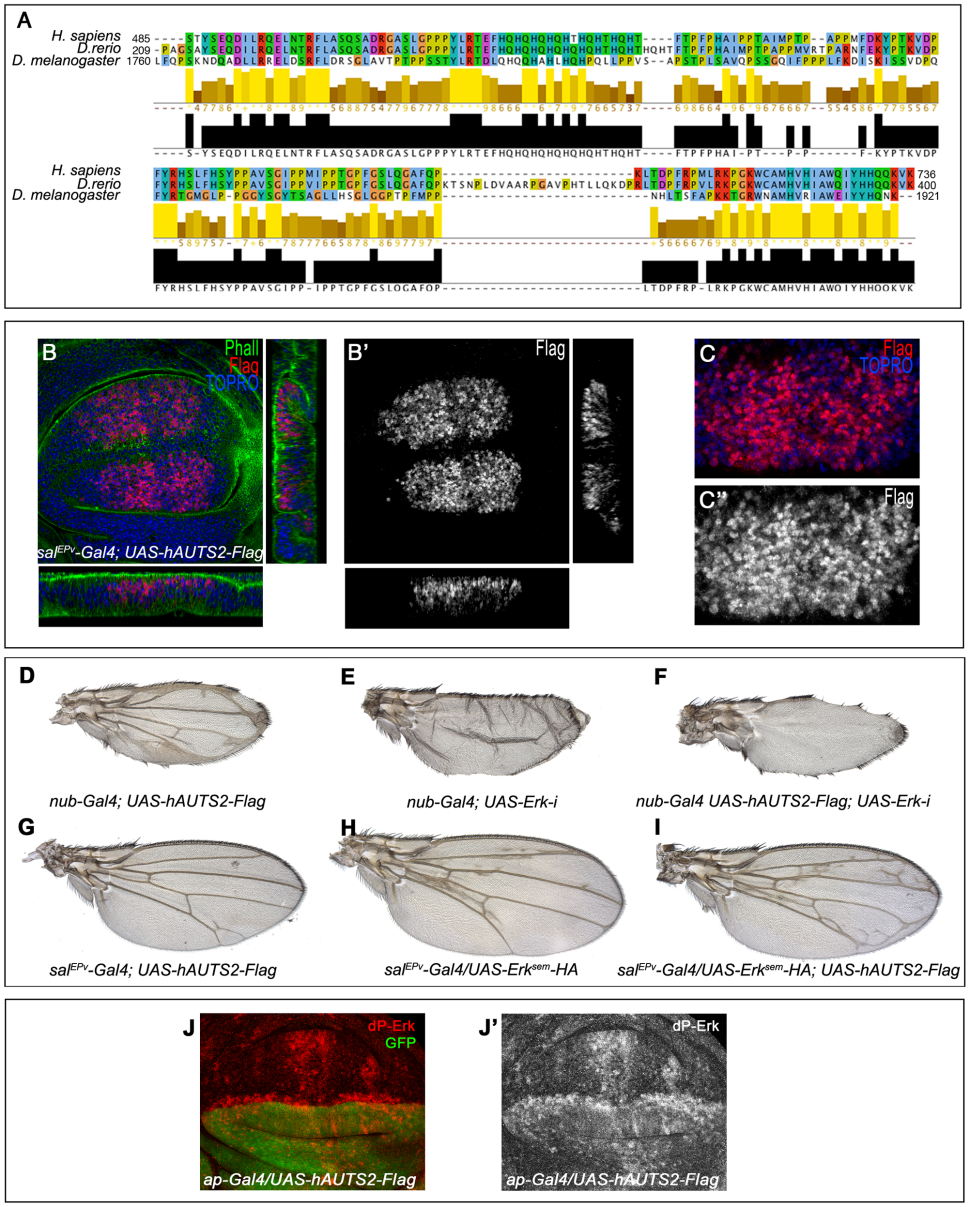
Subcelullar localization and phenotypes of human AUTS2 in flies. (A) Sequence conservation between human AUTS2 (*H. sapiens*; amino acids 485–736), Zebra fish AUTS2 (*D. renio*; amino acids 209–400) and Tay (*D. melanogaster*; amino acids 1760–1921). The colour code indicates the physicochemical properties of each amino acid (Jalview software) and the bars below each amino acid the identity in two or three species. (B–B′) Subcellular localization of AUTS2 (Flag red in B, white in B′) in wing discs of *sal^EPv^-Gal4/+; UAS-hAUTS2-Flag/+*. Below and to the right are the corresponding horizontal and transversal sections of the disc. The expression of F-actin is in green and To-Pro in blue. B′ is the red channel of B showing the accumulation of AUTS2. (C–C′) Higher magnification of the dorsal region of a *sal^EPv^-Gal4/+; UAS-hAUTS2-Flag/+* disc, showing the expression of AUTS2 (red in C, white in C″) and To-Pro (blue). The single red channel is shown in C′. (D–I) The phenotype of ectopic veins produced by the expression of AUTS2 in the entire wing blade and hinge (*nub-Gal4/UAS-hAUTS2-Flag*; D) is abolished when the expression of AUTS2 in accompanied with a reduction of Erk expression in *nub-Gal4/UAS-hAUTS2-Flag; UAS-Erk-i/+* flies (F), producing a loss of vein phenotype very similar to *nub-Gal4/UAS-Erk-i* flies (E). (G–I) Expression of AUTS2 in *sal^EPv^-Gal4/UAS-hAUTS2-Flag* flies (G) and expression of Erk^sem^ in *sal^EPv^-Gal4/UAS-Erk^sem^-HA* flies (H) causes weak phenotypes of ectopic veins that are enhanced when AUTS2 and Erk^sem^ are expressed together in *sal^EPv^-Gal4/UAS-Erk^sem^-HA; UAS-hAUTS2-Flag* flies (I). (J–J′) Third instar wing disc showing the expression of dP-Erk (red in J, white in J′) in *ap-Gal4/UAS-hAUTS2-Flag* wing discs. Note the difference in expression levels between dorsal (labelled in green in J) and ventral cells. J′ corresponds to the red channel of J.

*Drosophila* Tay and human AUTS2 are very different proteins in sequence and length, but they share a small 250 amino acid stretch with significant homology (Fig. 9A). Our deletion analysis of Tay indicates that this region is included in the smaller fragment of Tay that we found has biological activity and nuclear localization (C.M. and J.F.dC., unpublished results). We wanted to check whether AUTS2 expressed in flies was able to reproduce some of the effects observed in Tay over-expression conditions. A Flag-tagged form of AUTS2 expressed in the wing disc is localised exclusively in the nuclei (Fig. 9B–C), the same as Tay. Interestingly, the expression of AUTS2 in the wing leads to a phenotype of ectopic vein formation reminiscent to the consequence of Tay loss (Fig. 9D). The extra veins that develop in AUTS2 over-expression conditions depend on EGFR signalling, because they are eliminated when the expression of Erk is reduced (Fig. 9E–F). AUTS2 also enhances the formation of extra veins caused by the expression of Erk^sem^ (Fig. 9H–I), and causes an increase in the levels of activated Erk (*ap-Gal4/UAS-hAUTS2-Flag*; Fig. 9J–J′). These data suggest that AUTS2 is able to interact with some, but not all, targets of Tay, and raise the possibility that AUTS2 normal function in humans is related to the regulation of the Erk signalling pathway, albeit in an opposite manner as Tay.

## Discussion

Signalling by Erk in response to growth factors regulates growth, differentiation and survival of cells in a variety of developmental contexts [7], [52]–[53]. The extent and level of Erk activation relies on its phosphorylation state, which in turns regulates Erk subcellular localization and interactions with downstream effectors and other proteins [7], [10], [54]–[55]. Erk activation is transient, and failures in the mechanisms responsible for its inactivation can drive developmental defects and oncogenic transformations [10]. In this work we identified Tay as a novel nuclear component that interacts with Erk and is involved in the maintenance of appropriate levels of Erk activity.

### Tay is a nuclear protein that antagonize EGFR signalling

We have addressed the requirements and function of *tay* mostly in the wing disc, a convenient developmental system to analyse the contribution of signalling pathways to the regulation of organ size and pattern formation [56]. Tay was previously described as a protein that regulates locomotion and other neural aspects [38]–[39]. We have observed that changes in the level of EGFR signalling in the nervous system also cause locomotion defects (Molnar and de Celis, in preparation), which is indicative of a role of Tay in the regulation of EGFR signalling also in the nervous system. In the context of wing development and vein differentiation, the loss of *tay* results in the differentiation of extra veins in inter-vein territories. This phenotype is very similar to those obtained in conditions of excess of EGFR signalling, suggesting that Tay negatively regulates the activity or the response to this pathway. In addition, loss of *tay* also causes a reduction in the size of the wing blade, a phenotype that is not expected in a situation of excess of EGFR/ERK activity. This last result suggests that Tay might also have functions independent of its role in the regulation of EGFR signalling. The consequences of gain of Tay expression mostly indicate that the role of Tay is related to the modulation of EGFR signalling. Thus, excess of Tay expression in different imaginal discs results in phenotypes that can be attributed to loss of EGFR signalling, such as loss of veins and bristles [33], wing size reduction and failures in tarsal joint formation [57] and ommatidial differentiation (data not shown).

We further explore the relationships between Tay and EGFR signalling in genetic combinations in which the activity of the pathway is altered in backgrounds with modified levels of Tay expression. In all cases, we observed synergistic interactions between loss of *tay* and excess of EGFR, and between excess of *tay* and loss of EGFR activity. Furthermore, we notice that the extra veins differentiating in *tay* mutants require EGFR function, suggesting that Tay modulates EGFR signalling during vein formation. All together, the results of genetic combinations indicate that cells with lower levels of Tay become more sensitive to an increase in EGFR signalling, and that Tay over-expression prevents cells to acquire the level of EGFR signalling required for vein formation.

The negative effect of Tay on EGFR signalling is more directly visualised by considering the effects of Tay in Erk phosphorylation and in the expression of the EGFR/Erk targets genes *Dl* and *argos*. Thus, Tay over-expression strongly suppresses Erk phosphorylation and prevents the expression of *Dl* and *argos* in the developing veins. Conversely, in loss of *tay* conditions we detect an increase in the levels of phosphorylated Erk, which is accompanied by a moderate ectopic expression of *argos*. The extra-vein phenotype of loss of *tay* is not as extreme as the massive vein differentiation that occurs upon strong and constitutive activation of the EGFR pathway. In fact, *tay* mutant wings differentiate a similar pattern of extra veins as moderate increases in EGFR signalling caused by, for example, mutations in the *Mkp3* gene [58]. This suggest us that Tay primary function is to prevent increases in EGFR/Erk signalling in places where the pathway must be active but only at low levels. Thus, high levels of EGFR activity and dP-Erk accumulation are restricted to the presumptive veins in wild type third instar wing discs, but the pathway is also active at lower levels in the inter-veins, where it promotes cell proliferation and survival [28]. In *tay* or *Mkp3* mutant backgrounds, a fraction of these cells initiates the vein differentiation program, escaping the negative feed-back loops that maintain low dP-Erk levels and entering the positive feed-back loops that normally operate in vein territories through the regulation of *rhomboid* expression [59]. In this model, Tay would participate in a mechanism that favours Erk de-phosphorylation and its nuclear retention in an inactive form. This mechanism of Tay action is compatible with the effects of its over-expression, which essentially cause a failure to accumulate dP-Erk in vein territories, and consequently a loss of vein differentiation.

### Tay interacts with Erk and Mkp3

Signalling by Erk proteins in the nucleus is in part regulated by the rate of Erk nucleus/cytoplasm shuttling [60]. In the nucleus, signal termination involves Erk de-phosphorylation by nuclear phosphatases and also its sequestration away from cytoplasmic Erk kinases [61]. Because Erk does not contain nuclear localization nor export sequences, its subcellular localization relies on proteins acting as anchors [8]. We observed direct interactions between Tay and Erk and between Tay and Mkp3, and these interactions were also detected in immunoprecipitation experiments from embryo protein extracts. These data suggests that Tay could form part of protein complexes including both Erk and Mkp3 in the nucleus.

A direct interaction between Tay and Erk is also compatible with several observations regarding Tay stability and Erk subcellular localization. First, Erk and Erk^sem^ increase the accumulation of Tay in the nucleus, and do so independently of EGFR signalling, as neither Ras^V12^ nor Mkp3 over-expression modified Tay accumulation. Second, Tay over-expression prevents the accumulation of dP-Erk, whereas loss of Tay has the converse effect. Finally, Tay over-expression modifies Erk^sem^ subcellular localization, increasing the nucleus/cytoplasm ratio of Erk^sem^ accumulation. In this regard, it is worth noting that the expression of Ras^V12^ has the same effects on Erk^sem^ subcellular localization as the over-expression of Tay, as both Tay and Ras^V12^ increase the nuclear/cytoplasm ratio of Erk^sem^ accumulation. We notice that the effects of Tay on Erk localization are only manifest when we used the Erk^sem^ form. Because we also see that Erk^sem^ is not retained in the cytoplasm by Mkp3, we reason that Erk^sem^, liberated of cytoplasmic anchorage by Mkp3, is more sensitive to pathway activation and to the presence of other anchoring proteins, and that Tay might play this role in the nucleus.

We also observed a direct interaction between Tay and Mkp3. Mkp3 is a dual-specificity phosphatase that is predominantly localised in the cytoplasm, but it shuttles between the nucleus and cytoplasm and could play a role in translocating inactive Erk from the nucleus to the cytoplasm [8]. It is possible that Tay could promote the nuclear function of Mkp3, but in addition, Tay should also act independently of Mkp3 to promote Erk inactivation and retention, because Tay is able to down-regulate Erk activity in *Mkp3* mutant backgrounds.

Most of the Tay interacting region with Erk is localised to the C-terminal part of Tay, a 1000 amino acid long region that includes the domain of homology between Tay and human AUTS2. This fragment of Tay fails to interact with Mkp3, and is even more efficient than the full-length protein in its effects on Erk subcellular localization and in its antagonism on Erk signalling. Intriguingly, AUTS2 expressed in the wing disc also interferes with EGFR signalling, but it does so in an opposite manner to Tay or to the Tay C-terminal domain. We cannot extract many conclusions from the consequences of AUTS2 expression in the wing disc, but speculate that this protein retains some of its interactions with *Drosophila* Erk that might protect this protein from inactivation by nuclear phosphatases. Similarly, the effects of AUTS2 on *Drosophila* EGFR signalling are compatible with a role for this protein in the regulation of Erk activity in humans, and that this effects might underline the effects of zebrafish, murine and human mutations in the onset of neurological disorders.

From the analysis in the wing disc we conclude that Tay interacts with Erk in the nucleus, affecting its phosphorylation and promoting its nuclear retention. In this context, it is interesting to note that the free diffusion of human ERK2 is impeded within the nucleus, and that this limitation in mobility increases after ERK2 stimulation [6]. This has lead to postulate that ERK2 retention in the nucleus involves high-affinity interactions with unidentified low-mobility sites that are constitutively expressed [6]. We suggest that Tay could play such a role in vivo, acting as a nuclear anchor for Erk that facilitates its inactivation by nuclear phosphatases and its retention in an inactive state.

## Materials and Methods

### Genetic strains

We used the *Mkp3* allele *Mkp3^M76-R2b^* [58], and the deficiencies *EP-866^rev34^*, *EP-866^rev40^* and *Df(1)tay* (see below). We used the following Gal4 lines: *shv^3kpn^-Gal4* [62], *638-Gal4*, *nub-Gal4*, *sal^EPv^-Gal4* [63], *ap-Gal4*, *hh-Gal4*, *bs-Gal4*, *1348-Gal4*, *dll-Gal4*, *eye-Gal4* and *da-Gal4* [64]. We also used the UAS lines: *UAS-Ras^V12^* [65], *UAS-Raf^act^* [66], *UAS-Erk^sem^* [67], *UAS-Erk-HA* [55], *UAS-Erk^sem^-HA* [55], *UAS-rhomboid* [47], *UAS-EGFR*, *UAS-EGFR^DN^* [68] and *UAS-GFP* [69] and the P-GS lines *EP*-*M76* and *EP-866* [37]. We generated the following UAS lines: *UAS-tay-i*, *UAS-tay.FL-Flag*, *UAS-tay.1-Flag*, *UAS-tay.2-Flag*, *UAS-hAUTS2-Flag*, and *UAS-Mkp3-Myc*. We also used the RNA interference lines *UAS-Mkp3-i* (23911), *UAS-tay-i* (29021) and *UAS-rolled-i* (35641) from the VDRC Stock Center, and the lines *UAS-EGFR-i* (10079R-2) *and UAS-ras-i* (9375R-1) from NIG-Fly.

### Generation of *tay* alleles

*Df(1)tay:* We used the insertions *e03798* and *d06351* [70], which are separated by 15 Kb of DNA including *tay* and part of *CG16952*. Flipase (FLP)-induced recombination was induced by a daily 1 h heat shock at 37°C to the progeny of *e03798/d06351; hsFLP/+* females and FM7 males. Ten putative *e03798-d06351/FM7* offspring females were individually crossed to FM7 males, and after 3 days, were used to extract genomic DNA to determinate by PCR the existence of FLP recombination. The position of the flanking insertions *e03798* and *d06351* and the extent of the *tay* deficiency are described in Suppl. Fig. S2A.

*EP-866^rev40^ and EP-866^rev34^*: We used Δ2–3 as a source of transposase to mobilize the *EP-866* P-GS element. Males carrying both *EP-866* and Δ2–3 were crossed with *N^55e11^*/*FM7c* females. The offspring *EP-866* males with *white* phenotype were selected to make individual stocks. A complementation test was done to analyse the behaviour of these new alleles.

### Molecular identification of the *EP-866^rev40^* and *EP-866^rev34^* deficiencies

Fifty wild type (control) and homozygous *EP-866^rev40^* and *EP-866^rev34^* embryos were used to extract genomic DNA to identify by PCR the genomic region excised by the mobilization of the P-GS. We used the following primers: 5′GCCGTGGAAATGGACTCTG3′ and 5′TTGCTGCTGCTGGTGAAAT3′. The size of the amplified fragments was 3629pb in wild type embryos, 2373pb in *EP-866^rev34^* embryos and 932pb in *EP-866^rev40^* embryos. The size of the generated deficiencies was confirmed by sequencing the PCR fragments sub-cloned in the pGEM-T-Easy vector (Promega) confirming an excision of 1276pb in *EP-866^rev34^* and of 2717pb in EP*-866^rev40^*.

### Generation of FLP recombination clones

Homozygous *Df(1)tay*, *EP-866^rev40^* and *EP-866^rev34^* clones were induced in larvae of the following genotypes: *Df(1)tay f^36a^ FRT18A/FRT18A UbiGFP; hsFLP/+; EP-866^rev40^ f^36a^ FRT18A/FRT18A UbiGFP; hsFLP/+* and *EP-866^rev34^ f^36a^ FRT18A/FRT18A UbiGFP; hsFLP/+*, respectively. Homozygous *tay* mutant cells were recognized in the adult wing by the cellular marker *forked* (*f*) and in the wing disc by the absence of GFP.

### Generation of *tay* and *Mkp3* constructs

#### UAS-tay-i

The EST *LD22609* (DGRC) was used as a template to amplify a 700pb *tay* fragment using the following primers: 5′GCGCTCTAGAGCGGCAGCGATGGGCACAGTA3′ and 5′GCGCTCTAGAGCATGCGTAGCAGCAGGCGGCGGATAA3′. The amplified fragment was digested with the restriction enzyme *XbaI* (underlined sequence in the primers) and cloned into the pWIZ vector [71], previously digested with *AvrII*. The resulting plasmid was digested with *NheI* to clone the *tay* PCR fragment digested with *XbaI*. The orientation of both *XbaI* fragments cloned into pWIZ was checked to confirm an inverted position.

#### Tay-Flag constructs

In order to generate epitope tagged Tay proteins, the cDNA clone *LD22609* was used as a template to amplify *tay* fragments using the following primers: Tay.FL 5′CACCATGGACACATCAAATGCCAGCGC3′ and 5′TCGACTGGGCGCCACCGATG3′; Tay.1 5′CACCATGGACACATCAAATGCCAGCGC3′ and 5′TGGAGGCAGGTATGACCCGTG3′ and Tay.2 5′CACCATGTCGCAGAATCAGCCAATGGTT3′ and 5′TCGACTGGGCGCCACCGATG3′. These PCR products were directionally subcloned into pENTR/D-TOPO (Invitrogen). For generating the C-terminal-Flag-tagged fusion proteins, we used the LR Clonase II reaction with Tay-pENTR/D-TOPO clones and the pTWF (3XFlag-tag at the C-terminal) vector for expression in vivo in Gal4-expressing cells following the instructions from Invitrogen.

#### Mkp3-Myc construct

The cDNA clone *SD06439* (DGCR) was used as a template to amplify the *Mkp3* cDNA using the following primers: 5′CACCATGCCAGAAACGGAGCACGAG3′ and 5′TTTAAGACCCGTGTCCGACGG3′. This PCR product was directionally cloned into pENTR/D-TOPO (Invitrogen). For generating the C-terminal-Myc-tagged fusion proteins, we used the LR Clonase II reaction with Mkp3-pENTR/D-TOPO and the pTWM (6XMyc-tag at the C-terminal) vector for expression in vivo in Gal4-expressing cells following the instructions from Invitrogen.

#### UAS-hAUTS2-Flag

The Full Length clone IRAKp961I02133Q (imaGenes) was used as a template to amplify the human *AUTS2* cDNA using the following primers: 5′CACCATGGATGGCCCGACGCGGGGC3′ and 5′TCGGGCCTCGATATCCTTCAG3′. These PCR products were directionally cloned into pENTR/D-TOPO (Invitrogen). For generating the C-terminal-Flag-tagged fusion proteins, we used the LR Clonase II reaction with hAUTS2-pENTR/D-TOPO and the pTWF (3XFlag-tag at the C-terminal) vector for expression in vivo in Gal4-expressing cells following the instructions from Invitrogen.

### Generation of Tay antiserum

#### Protein expression and purification

Fusion protein containing amino acids 1756–2049 of Tay was generated using *LD22609* as template and the following primer pair: 5′GCGCGGATCCTCTGCAGAATCGCTTTTTCAG3′ and 5′GCGCATGAATTCCTCACACTGCGGTTCCAATATGACT3′ containing *BamHI* and *EcoRI* restriction sites respectively (underlined sequence). The amplified fragment was digested with the restriction enzymes *BamHI* and *EcoRI*, cloned in the *BamHI*-*EcoRI* site of the Gluthatione-S-Transferase (GST) gene fusion pGEX-2T (Promega) vector and transformed in *E. coli* BL21 (DE3). Selected clones were verified by sequencing.

#### Antibody generation

The GST-Tay_1756–2049_ protein was expressed in *E. coli* BL21 (DE3) and purified using Glutathione Sepharose 4B (GE Healthcare). The anti-Tay antibody was prepared by immunizing rats and guinea pigs with purified GST-Tay_1756–2049_ following conventional procedures.

### Immunohistochemistry

We used the rabbit antibodies: anti-phospho-Histone3, anti-activated Cas3 and anti-diphosphorylated ERK1&2 (Cell Signalling). We also use the mouse monoclonal antibodies: anti-c-Myc 9E10 (Santa Cruz Biotechnology), anti-HA 12CA5 (Sigma), anti-FlagM2 (Sigma), anti-βGal (Promega), and anti-FasIII, anti-Dl and anti-Arm from the Hybridoma Bank at University of Iowa (Iowa City, IA). Alexa Fluor secondary antibodies (used at 1∶200 dilution) were from Invitrogen. To stain the nuclei we used To-Pro and to stain F-actin we used Alexa Fluor Phalloidin, from Invitrogen. Imaginal wing discs were dissected, fixed, and stained as described in [72]. Confocal images were taken in a LSM510 confocal microscope (Zeiss). *In situ* hybridization with the *tay* probe were carried out as described [72]. We used the cDNA *LD22609* as template to synthesize the *tay* probe. The quantification of Erk^sem^ nuclear and cytoplasmic staining was carried out in Z-sections taken from 6 proximo-distal planes of 6 discs of each genotype along the length of the epithelium with the program ImageJ.

### Pull-down assays

The fusion proteins Mkp3-GST and Erk-GST and the GST protein (negative control) were expressed in *E. coli* BL21 (DE3), using the constructs pGEX2TK-DMkp3 and pGEX4T1-DErk [73] and the vector pGEX2T, respectively, and were purified using Glutathione Sepharose 4B (GE Healthcare). The complete Tay protein was generated from the cDNA *LD22609* using the TNT T7 Coupled Reticulocyte Lysate System (Promega) and radiolabeled with S^35^-Met. The pull-down assay was performed incubating over-night at 4°C the same amount of GST or GST fusion proteins bound to Glutathione Sepharose4B with in vitro translated Tay. After centrifugation and washes the proteins were resolved by 6% SDS/PAGE and the existence of pull-down proteins was analysed by autoradiography. The pulldown experiments were repeated five times with the same results.

### Western blot and immunoprecipitation

#### Embryos lysate

Embryos were collected 24 hours after egg laying, washed in a 0.7% NaCl + 0.04% Triton X-100 solution, de-corionated in 50% bleach and frozen at −80°C. Embryos were homogenised in immunoprecipitation (IP) buffer (40 mM Tris-HCl, 200 mM NaCl, 1% NP-40, 0.1% SDS, 0.5%DOC, 6 mM EDTA, 6 mM EGTA, 100 µM PMSF and complete EDTA-free protease inhibitor cocktail tablet (Roche)) and incubated 2 h at 4°C. Lysates were clarified by centrifugation. Protein concentration in these lysates was determined using the Lowry-Peterson protocol.

#### Immunoprecipitation

50 µg and 2 mg of total protein from each lysate were used to assess protein expression levels (input) and for the immunoprecipitation reactions, respectively. The immunoprecipitation reactions were performed by incubating the lysates with 1 mg/ml BSA, 10% Glycerol and specific antibodies for Mkp3-Myc (15 µl of anti-Myc agarose, Santa Cruz Biotechnology), for Erk^sem^-HA (15 µl of anti-HA agarose, Santa Cruz Biotechnology) and for Tay.FL-Flag and Tay.2-Flag (15 µl of anti-FlagM2 agarose, Sigma) at 4°C for 12–16 h. Erk^sem^ is a mutated form of Erk that shows less sensitivity to Mkp3 [50].

#### Immunoblotting

Embryo lysates or immunoprecipitated complexes were resolved by 7% SDS-PAGE and proteins were transferred to nitrocellulose membranes using a semi-dry blotting apparatus (BioRad). Erk^sem^ and Mkp3 proteins were detected by incubating with anti-HA (HA 12CA5, Hybridome Bank) and anti-Myc (c-Myc 9E10, Santa Cruz Biotechnology) monoclonal antibodies, respectively. Tay proteins were detected by incubating with anti-Tay guinea pig serum. Immunoblots were developed using IR680 and 800 labelled antibodies (Li-Cor) with the Odyssey Infrared Imaging System (Li-Cor). The immunoprecipitation experiments for Tay.FL and Mkp3 were repeated 7 times, for Tay.FL and Erk^sem^ 5 times, for Tay.2 and Mkp3 5 times and for Tay.2 and Erk^sem^ 4 times.

## Supporting Information

Figure S1 Phenotypes of *EP-866/Gal4* combinations in the wing, thorax and leg. (A–B) Wild type adult tarsus (A) and thorax (B). (C–D) Adult tarsus and notum of *EP-866/+; dll-Gal4/+* flies showing fusion and disorganization of the joints (C) and loss of macro- and microchaetae in the thorax of *EP-866/+; ap-Gal4/+* (D). (E–E′) Wild type *sal^EPv^-Gal4/UAS-GFP* third instar wing disc showing the expression of GFP (green) and Phospho-Histone3 (PH3; red). E′ is the red channel of E. The mitotic index in the *sal^EPv^-Gal4/UAS-GFP* domain of expression is 0.0014 (sd:0.0002, n = 10). (F–F′) Third instar wing imaginal disc of *EP-866; sal^EPv^-Gal4/UAS-GFP* flies showing a reduction of cells expressing the mitotic marker PH3 (PH3 in red, GFP in green). F′ is the red channel of F. The mitotic index in the *EP-866; sal^EPv^-Gal4/UAS-GFP* domain of expression is 0.0007 (sd:0.00026, n = 10). (G–G′) Third instar wing imaginal disc of *EP-866/+; nub-Gal4/+* showing a complete absence of cell death (Cas3 in green, Armadillo in red). (H–M) Adult wings showing reduction of wing size and defects in vein differentiation in flies over-expressing Tay during pupal development (*EP-866/+; 1348-Gal4/+*; H), in larval and pupal stages (*EP-866/+; bs-Gal4/+*; I), in the posterior comparent of the wing disc (*EP-866/+; hh-Gal4/+*; J) and in the central domain of the wing disc at different dosages (*EP-866/+; sal^EPv^-Gal4/+* (K) and *EP-866; sal^EPv^-Gal4/sal^EPv^-Gal4* females (L) and males (M).
(TIF)

Figure S2 Genetic tools used to analyse the loss of function of *tay*. (A) Map of the *tay* genomic region indicating the intro-exon structure of the transcript, the extent of the two deficiencies induced by imprecise transposition (*EP-866^Rev34^* and *EP-866^Rev40^*), the position of the Piggy-Bac elements used to generate the deletion *Df(1)tay* (red triangles) and the position of the *EP-866* insertion (black triangle). (B) Schematic representation of the Tay protein showing in red the 300 amino acid fragment used to generate the polyclonal antibody (1757–2049.GST). (C–C′) Clone of *Df(1)tay* cells (black) and twin spot (intense red), showing that the protein is absent in the clone. Clones were induced in larvae of *Df(1)tay* FRT18A*/FRT18A tub-GFP; hs-FLP32/+* genotype, and the red channel showing Tay expression is shown in C′. (D–E) Examples of Tay expression in embryos used to make protein extracts of *da-Gal4/UAS-tay.FL-Flag; UAS-Erk^sem^-HA* (D) and *da-Gal4/UAS-tay.FL-Flag; UAS-Mkp3-Myc* (E) genotypes.
(TIF)

Figure S3 Expression of dP-Erk in the embryonic tracheal pits and leg imaginal discs. (A–B′). Expression of dP-Erk in stage 11 wild type embryos (A–A′) and in stage 11 *EP-866^rev34^* embryos (B–B′), showing increased dP-Erk accumulation in *tay* mutants. A′ and B′ are higher magnification pictures. (C–D′) Expression of dP-Erk in wild type leg imaginal discs (C–C′) and *dll-Gal4/UAS-tay* leg discs (D–D′). The leg discs over-expressing *tay* display a generalised reduction of dP-Erk and loss of distal segments. C′ and D′ are the single red channels of C and D, respectively. (E–G) Expression of Tay during embryonic development. Prominent expression is detected in the central nervous system from stage 13 onwards (G).
(TIF)

Figure S4 Subcellular localization of Mkp3 and Erk in Tay over-expression conditions. High magnification confocal pictures of the dorsal compartment of third instar wing discs. (A) Expression of GFP (green), Mkp3-Myc (red) and To-Pro (blue) in discs of *sal^EPv^-Gal4 UAS-GFP/+; UAS-Mkp3-Myc/+* genotype. (A′–A′″) Single channels for GFP (A′), Mkp3-Myc (A″) and To-Pro (A′″). Mkp3 is mostly localised in the cytoplasm, but a weak signal is also detected in the nucleus (A″). (B) Expression of Tay (green), Mkp3-Myc (red) and To-Pro (blue) in discs of *sal^EPv^-Gal4 UAS-GFP/+; UAS-* Mkp3-Myc*/UAS-tay.FL-Flag* genotype. (B′–B′″) Single channels of B showing the nuclear localization of Tay (B′) and the preferential cytoplasmic localization of Mkp3 (B″) in cells over-expressing these proteins. (C) Expression of GFP (green), Erk-HA (red) and To-Pro (blue) in discs of *sal^EPv^-Gal4 UAS-GFP/+; UAS-Erk-HA/+* genotype. (C′–C′″) Single channels of C showing the nucleus-cytoplasmic localization of Erk-HA (C″). (D) Expression of Tay (green), Erk-HA (red) and To-Pro (blue) in discs of *sal^EPv^-Gal4/+; UAS-Erk-HA/UAS-tay.FL-Flag* genotype. (D′–D′″) Single channels of D showing that the nuclear localization of Tay (D′) and the nucleus-cytoplasmic localization of Erk (D″) are not altered when these proteins are over-expressed in the same cells. (E) Expression of GFP (green), Erk^sem^-HA (red) and To-Pro (blue) in discs of *sal^EPv^-Gal4 UAS-GFP/+; UAS-Erk^sem^-HA/+* genotype. (E′–E′″) Single channels of E showing the nucleus-cytoplasmic localization of Erk^sem^-HA (E″). (F) Expression of Tay (green), Erk^sem^-HA (red) and To-Pro (blue) in discs of *sal^EPv^-Gal4/+; UAS-Erk^sem^-HA/UAS-tay.FL-Flag* genotype. (F′–F′″) Single channels of F showing that Erk^sem^-HA (F″) is now also accumulated in the nucleus in Tay over-expression conditions.
(TIF)

Figure S5 Subcellular localization of Erk and Erk^sem^ in Tay and Ras^V12^ over-expression conditions. (A–A″) Erk protein (HA, red in A; white in A′) is localized both in the nuclei and cytoplasm in cells over-expressing Ras^V12^ in *sal^EPv^-Gal4/UAS-Erk-HA; UAS-Ras^V12^/+* discs. (B–B″) This localization does not change when Tay is also over-expressed (*sal^EPv^-Gal4/UAS-Erk-HA; UAS-Ras^V12^/UAS-tay.FL-Flag*). (C–C″) Erk^sem^ protein (HA, red in C; white in C′) is localized predominantly in the nuclei in cells over-expressing Ras^V12^ in *sal^EPv^-Gal4/UAS-Erk^sem^-HA; UAS-Ras^V12^/+* discs. (D–D″) This localization does not change when Tay is also over-expressed (*sal^EPv^-Gal4/UAS-Erk^sem^-HA; UAS-Ras^V12^/UAS-tay.FL-Flag*).
(TIF)

Figure S6 Subcellular localization of Tay and its C-terminal and N-terminal fragments in Ras^V12^ over-expression conditions. (A–A″) Nuclear localization of Tay (Flag, red in A; white in A′–A″) when over-expressed in the central domain of the wing imaginal discs (*sal^EPv^-Gal4/UAS-tay.FL-Flag*). (A″) Higher magnification of the dorsal side of the disc shown in A–A′. (B–B″) Tay localization does not change when Ras^V12^ is expressed in the same cells (*sal^EPv^-Gal4/UAS-tay.FL-Flag*; *UAS-Ras^V12^/+*). (B″) Higher magnification of the dorsal side of the disc shown in B–B′. (C–C″) Cytoplasmic localization of the N-terminal fragment of Tay when over-expressed in the central domain of wing imaginal discs (*sal^EPv^-Gal4/UAS-tay.1-Flag*). (C″) Higher magnification of the dorsal side of the disc shown in C–C′. (D–D″) The cytoplasmic localization of the N-terminal fragment of Tay does not change when Ras^V12^ is expressed in the same cells (*sal^EPv^-Gal4/UAS-tay.1-Flag*; *UAS-Ras^V12^/+*). (D″) Higher magnification of the dorsal side of the disc shown in D–D′. (E–E″) Nuclear localization of the C-terminal fragment of Tay (Tay.2) over-expressed in the central domain of wing imaginal discs (*sal^EPv^-Gal4/UAS-tay.2-Flag*). (E″) Higher magnification of the dorsal side of the disc shown in E–E′. (F–F″) The nuclear localization of Tay.2 does not change when Ras^V12^ is expressed in the same cells (*sal^EPv^-Gal4/UAS-tay.2-Flag*; *UAS-Ras^V12^/+*). (F″) Higher magnification of the dorsal side of the disc shown in F–F′. In all panels Tay-Flag expression is in red and To-Pro in blue. Orthogonal sections are show to the right of each panel.
(TIF)

Figure S7 Changes in Tay and Tay.2 accumulation in response to Erk^sem^. (A–B) Expression of Tay in control *EP-866*; *sal^EPv^-Gal4 UAS-GFP/+* (A) and in wing discs over-expressing Erk^sem^ (*EP-866*; *sal^EPv^-Gal4 UAS-*ERK^sem^ */+*; B). (C–D) Expression of Tay.2 in control *UAS-tay.2-Flag*; *sal^EPv^-Gal4 UAS-GFP/+* (C) and in wing discs over-expressing also Erk^sem^ (*UAS-tay.2-Flag*; *sal^EPv^-Gal4 UAS-*Erk^sem^ */+*; D). Wing discs were fixed, stained and visualised using the same conditions. Note the higher levels of Tay and Tay.2 accumulation in the presence of Erk ^sem^.
(TIF)
