# Supplementary figures and images for: Tay Bridge Is a Negative Regulator of EGFR Signalling and Interacts with Erk and Mkp3 in the Drosophila melanogaster Wing
Source: PLoS Genet. 2013 Dec 12;9(12):e1003982. doi: 10.1371/journal.pgen.1003982 (PMC3861119; doi:10.1371/journal.pgen.1003982)

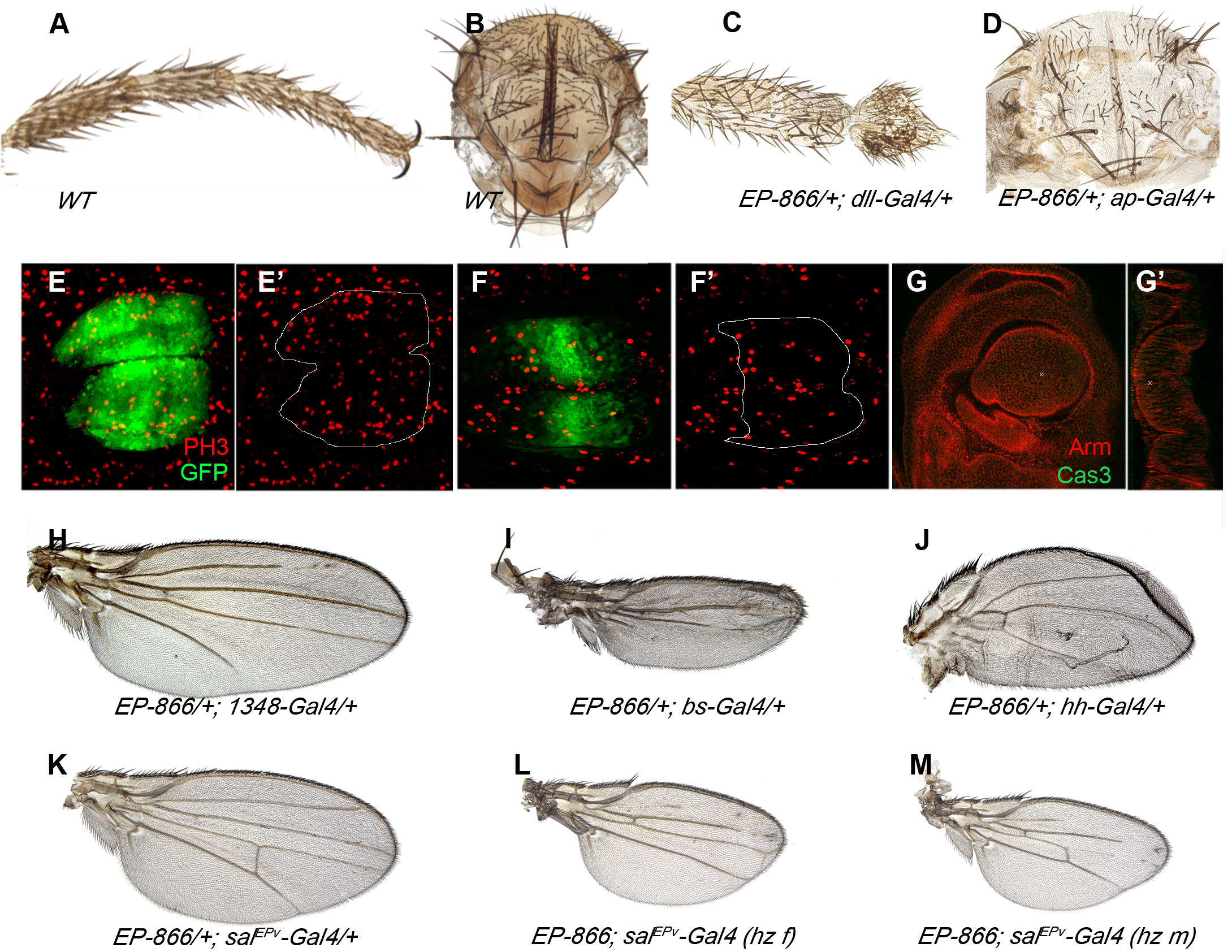

Supplement: Figure S1 — Phenotypes of EP-866/Gal4 combinations in the wing, thorax and leg. (A–B) Wild type adult tarsus (A) and thorax (B). (C–D) Adult tarsus and notum of EP-866/+; dll-Gal4/+ flies showing fusion and disorganization of the joints (C) and loss of macro- and microchaetae in the thorax of EP-866/+; ap-Gal4/+ (D). (E–E′) Wild type salEPv-Gal4/UAS-GFP third instar wing disc showing the expression of GFP (green) and Phospho-Histone3 (PH3; red). E′ is the red channel of E. The mitotic index in the salEPv-Gal4/UAS-GFP domain of expression is 0.0014 (sd:0.0002, n = 10). (F–F′) Third instar wing imaginal disc of EP-866; salEPv-Gal4/UAS-GFP flies showing a reduction of cells expressing the mitotic marker PH3 (PH3 in red, GFP in green). F′ is the red channel of F. The mitotic index in the EP-866; salEPv-Gal4/UAS-GFP domain of expression is 0.0007 (sd:0.00026, n = 10). (G–G′) Third instar wing imaginal disc of EP-866/+; nub-Gal4/+ showing a complete absence of cell death (Cas3 in green, Armadillo in red). (H–M) Adult wings showing reduction of wing size and defects in vein differentiation in flies over-expressing Tay during pupal development (EP-866/+; 1348-Gal4/+; H), in larval and pupal stages (EP-866/+; bs-Gal4/+; I), in the posterior comparent of the wing disc (EP-866/+; hh-Gal4/+; J) and in the central domain of the wing disc at different dosages (EP-866/+; salEPv-Gal4/+ (K) and EP-866; salEPv-Gal4/salEPv-Gal4 females (L) and males (M). (TIF) [file pgen.1003982.s001.tif]

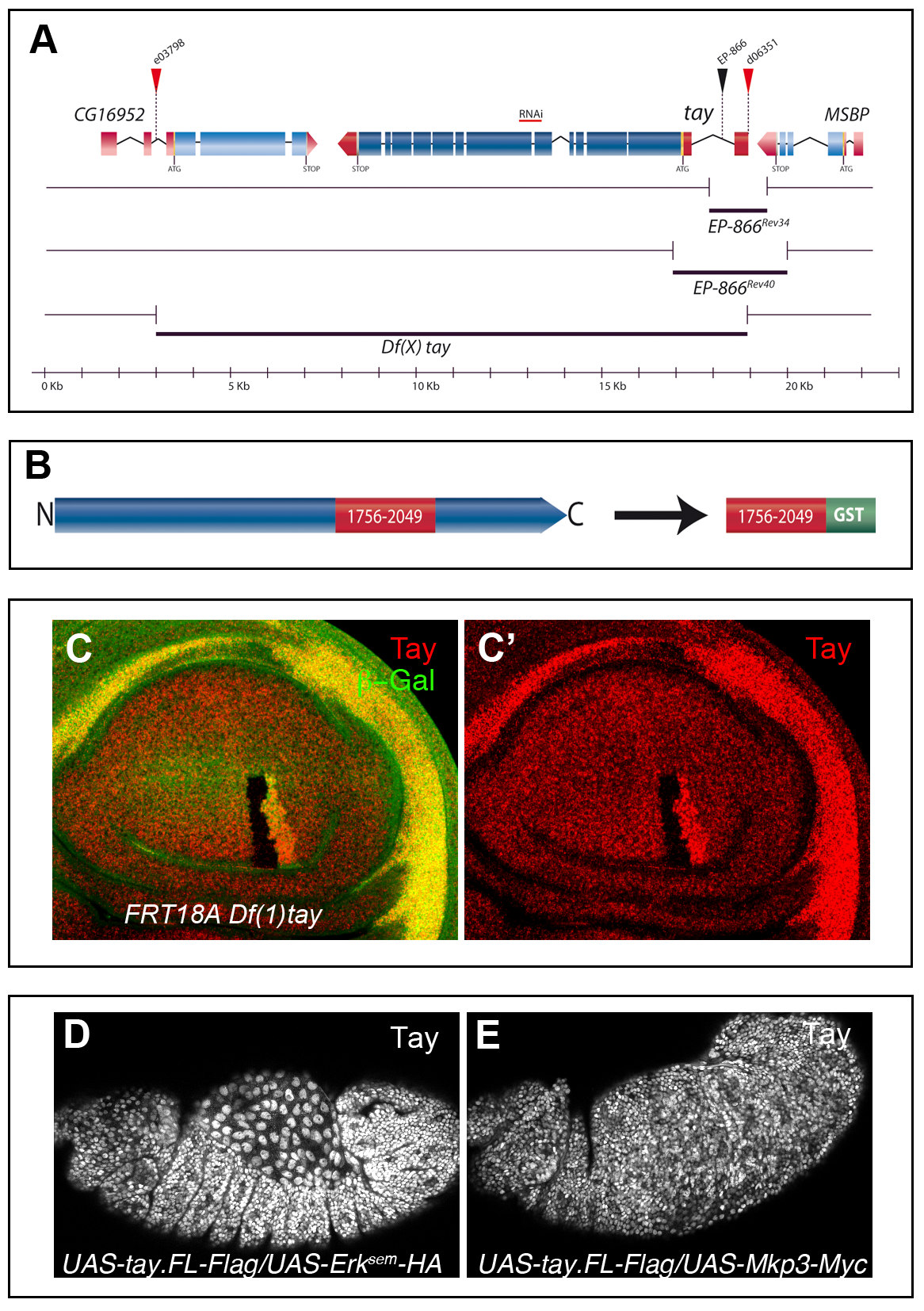

Supplement: Figure S2 — Genetic tools used to analyse the loss of function of tay. (A) Map of the tay genomic region indicating the intro-exon structure of the transcript, the extent of the two deficiencies induced by imprecise transposition (EP-866Rev34 and EP-866Rev40), the position of the Piggy-Bac elements used to generate the deletion Df(1)tay (red triangles) and the position of the EP-866 insertion (black triangle). (B) Schematic representation of the Tay protein showing in red the 300 amino acid fragment used to generate the polyclonal antibody (1757–2049.GST). (C–C′) Clone of Df(1)tay cells (black) and twin spot (intense red), showing that the protein is absent in the clone. Clones were induced in larvae of Df(1)tay FRT18A/FRT18A tub-GFP; hs-FLP32/+ genotype, and the red channel showing Tay expression is shown in C′. (D–E) Examples of Tay expression in embryos used to make protein extracts of da-Gal4/UAS-tay.FL-Flag; UAS-Erksem-HA (D) and da-Gal4/UAS-tay.FL-Flag; UAS-Mkp3-Myc (E) genotypes. (TIF) [file pgen.1003982.s002.tif]

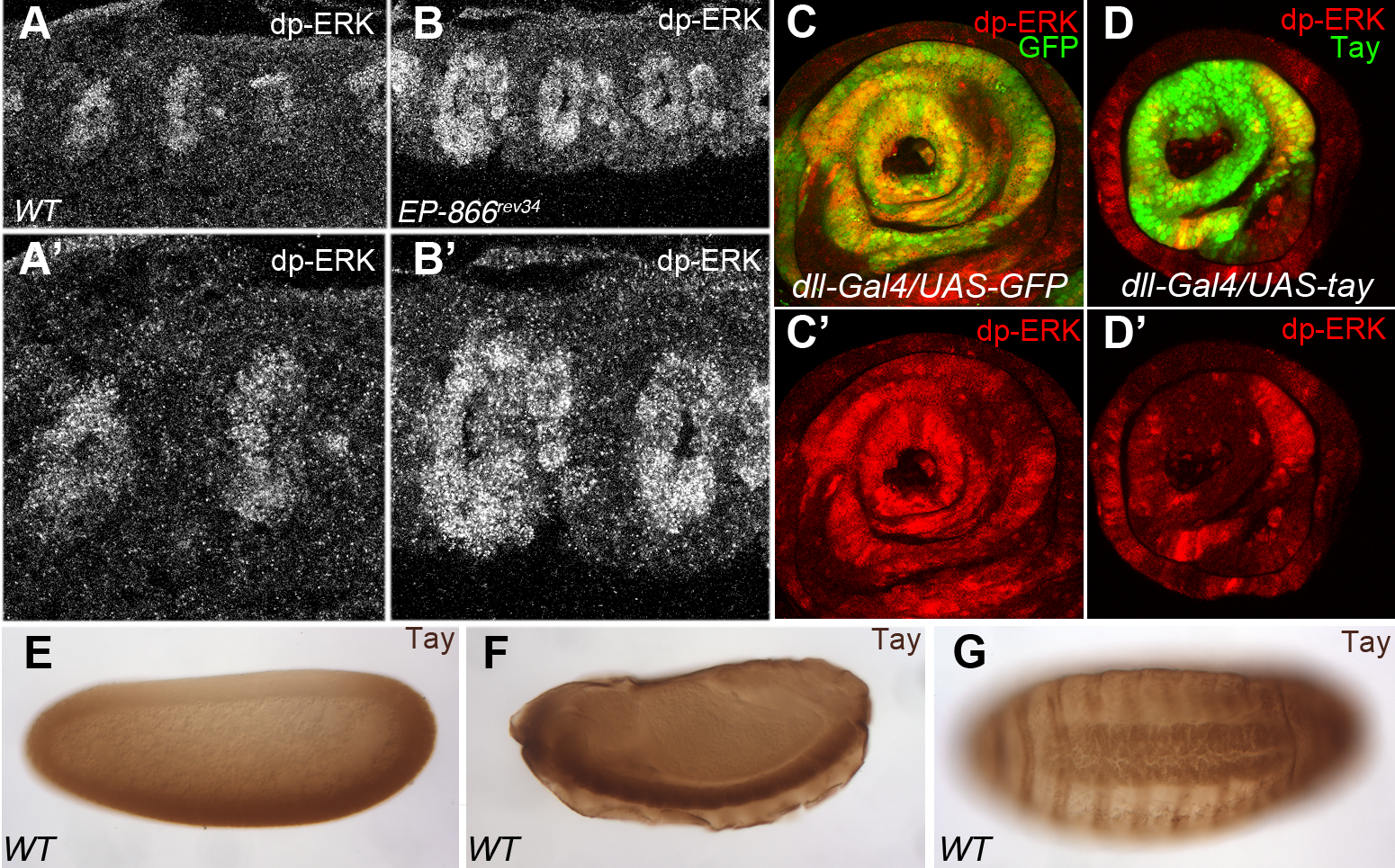

Supplement: Figure S3 — Expression of dP-Erk in the embryonic tracheal pits and leg imaginal discs. (A–B′). Expression of dP-Erk in stage 11 wild type embryos (A–A′) and in stage 11 EP-866rev34 embryos (B–B′), showing increased dP-Erk accumulation in tay mutants. A′ and B′ are higher magnification pictures. (C–D′) Expression of dP-Erk in wild type leg imaginal discs (C–C′) and dll-Gal4/UAS-tay leg discs (D–D′). The leg discs over-expressing tay display a generalised reduction of dP-Erk and loss of distal segments. C′ and D′ are the single red channels of C and D, respectively. (E–G) Expression of Tay during embryonic development. Prominent expression is detected in the central nervous system from stage 13 onwards (G). (TIF) [file pgen.1003982.s003.tif]

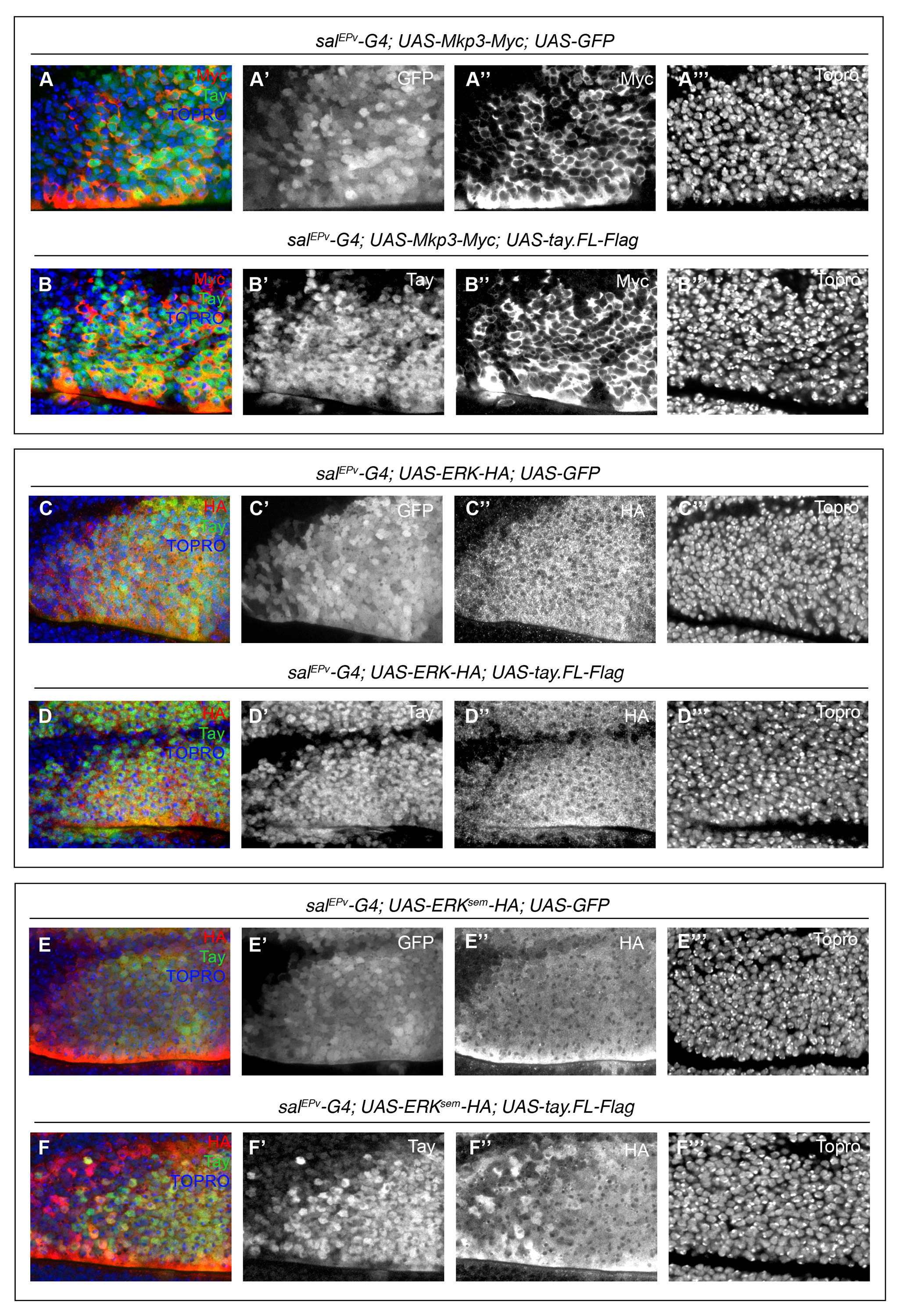

Supplement: Figure S4 — Subcellular localization of Mkp3 and Erk in Tay over-expression conditions. High magnification confocal pictures of the dorsal compartment of third instar wing discs. (A) Expression of GFP (green), Mkp3-Myc (red) and To-Pro (blue) in discs of salEPv-Gal4 UAS-GFP/+; UAS-Mkp3-Myc/+ genotype. (A′–A′″) Single channels for GFP (A′), Mkp3-Myc (A″) and To-Pro (A′″). Mkp3 is mostly localised in the cytoplasm, but a weak signal is also detected in the nucleus (A″). (B) Expression of Tay (green), Mkp3-Myc (red) and To-Pro (blue) in discs of salEPv-Gal4 UAS-GFP/+; UAS- Mkp3-Myc/UAS-tay.FL-Flag genotype. (B′–B′″) Single channels of B showing the nuclear localization of Tay (B′) and the preferential cytoplasmic localization of Mkp3 (B″) in cells over-expressing these proteins. (C) Expression of GFP (green), Erk-HA (red) and To-Pro (blue) in discs of salEPv-Gal4 UAS-GFP/+; UAS-Erk-HA/+ genotype. (C′–C′″) Single channels of C showing the nucleus-cytoplasmic localization of Erk-HA (C″). (D) Expression of Tay (green), Erk-HA (red) and To-Pro (blue) in discs of salEPv-Gal4/+; UAS-Erk-HA/UAS-tay.FL-Flag genotype. (D′–D′″) Single channels of D showing that the nuclear localization of Tay (D′) and the nucleus-cytoplasmic localization of Erk (D″) are not altered when these proteins are over-expressed in the same cells. (E) Expression of GFP (green), Erksem-HA (red) and To-Pro (blue) in discs of salEPv-Gal4 UAS-GFP/+; UAS-Erksem-HA/+ genotype. (E′–E′″) Single channels of E showing the nucleus-cytoplasmic localization of Erksem-HA (E″). (F) Expression of Tay (green), Erksem-HA (red) and To-Pro (blue) in discs of salEPv-Gal4/+; UAS-Erksem-HA/UAS-tay.FL-Flag genotype. (F′–F′″) Single channels of F showing that Erksem-HA (F″) is now also accumulated in the nucleus in Tay over-expression conditions. (TIF) [file pgen.1003982.s004.tif]

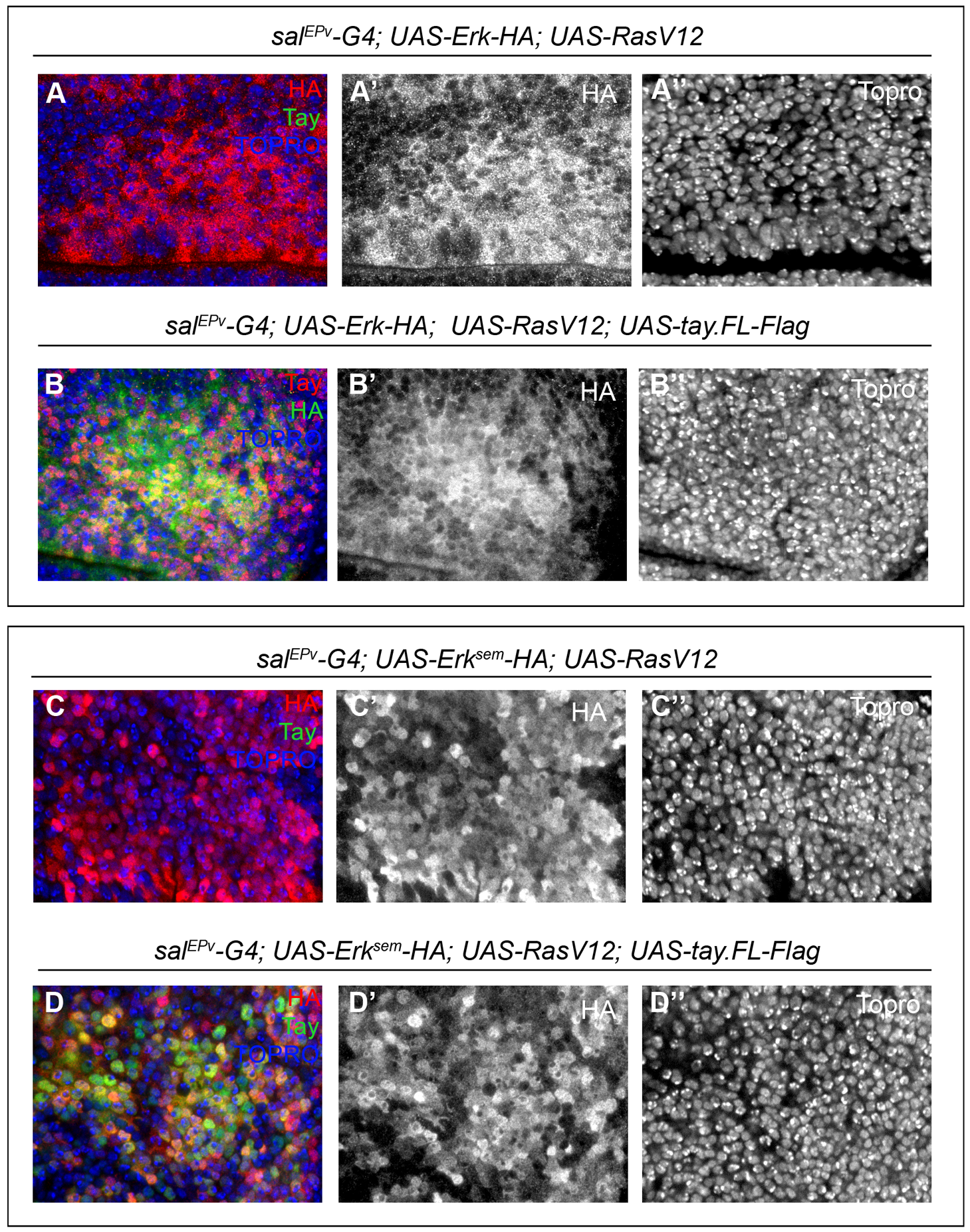

Supplement: Figure S5 — Subcellular localization of Erk and Erksem in Tay and RasV12 over-expression conditions. (A–A″) Erk protein (HA, red in A; white in A′) is localized both in the nuclei and cytoplasm in cells over-expressing RasV12 in salEPv-Gal4/UAS-Erk-HA; UAS-RasV12/+ discs. (B–B″) This localization does not change when Tay is also over-expressed (salEPv-Gal4/UAS-Erk-HA; UAS-RasV12/UAS-tay.FL-Flag). (C–C″) Erksem protein (HA, red in C; white in C′) is localized predominantly in the nuclei in cells over-expressing RasV12 in salEPv-Gal4/UAS-Erksem-HA; UAS-RasV12/+ discs. (D–D″) This localization does not change when Tay is also over-expressed (salEPv-Gal4/UAS-Erksem-HA; UAS-RasV12/UAS-tay.FL-Flag). (TIF) [file pgen.1003982.s005.tif]

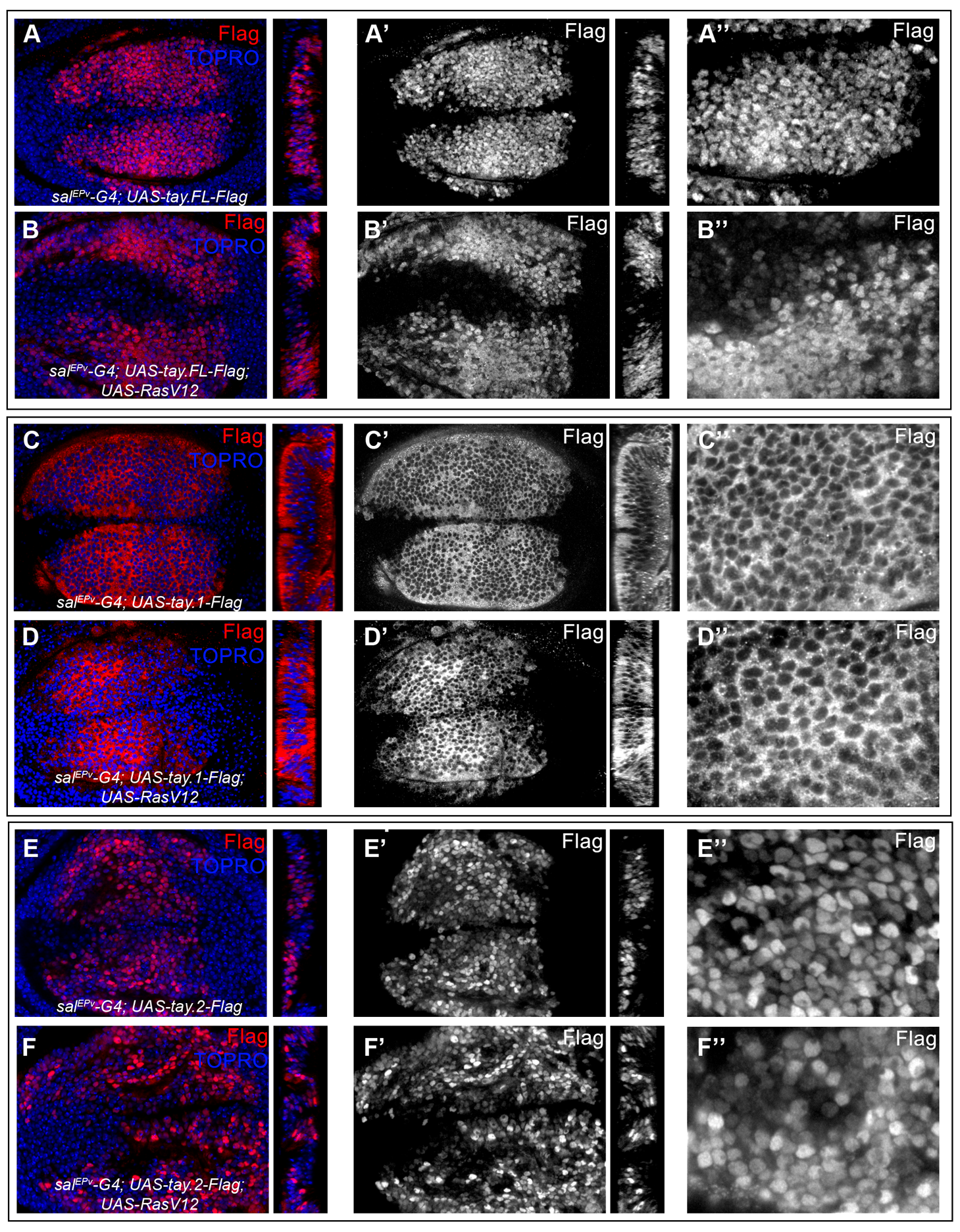

Supplement: Figure S6 — Subcellular localization of Tay and its C-terminal and N-terminal fragments in RasV12 over-expression conditions. (A–A″) Nuclear localization of Tay (Flag, red in A; white in A′–A″) when over-expressed in the central domain of the wing imaginal discs (salEPv-Gal4/UAS-tay.FL-Flag). (A″) Higher magnification of the dorsal side of the disc shown in A–A′. (B–B″) Tay localization does not change when RasV12 is expressed in the same cells (salEPv-Gal4/UAS-tay.FL-Flag; UAS-RasV12/+). (B″) Higher magnification of the dorsal side of the disc shown in B–B′. (C–C″) Cytoplasmic localization of the N-terminal fragment of Tay when over-expressed in the central domain of wing imaginal discs (salEPv-Gal4/UAS-tay.1-Flag). (C″) Higher magnification of the dorsal side of the disc shown in C–C′. (D–D″) The cytoplasmic localization of the N-terminal fragment of Tay does not change when RasV12 is expressed in the same cells (salEPv-Gal4/UAS-tay.1-Flag; UAS-RasV12/+). (D″) Higher magnification of the dorsal side of the disc shown in D–D′. (E–E″) Nuclear localization of the C-terminal fragment of Tay (Tay.2) over-expressed in the central domain of wing imaginal discs (salEPv-Gal4/UAS-tay.2-Flag). (E″) Higher magnification of the dorsal side of the disc shown in E–E′. (F–F″) The nuclear localization of Tay.2 does not change when RasV12 is expressed in the same cells (salEPv-Gal4/UAS-tay.2-Flag; UAS-RasV12/+). (F″) Higher magnification of the dorsal side of the disc shown in F–F′. In all panels Tay-Flag expression is in red and To-Pro in blue. Orthogonal sections are show to the right of each panel. (TIF) [file pgen.1003982.s006.tif]

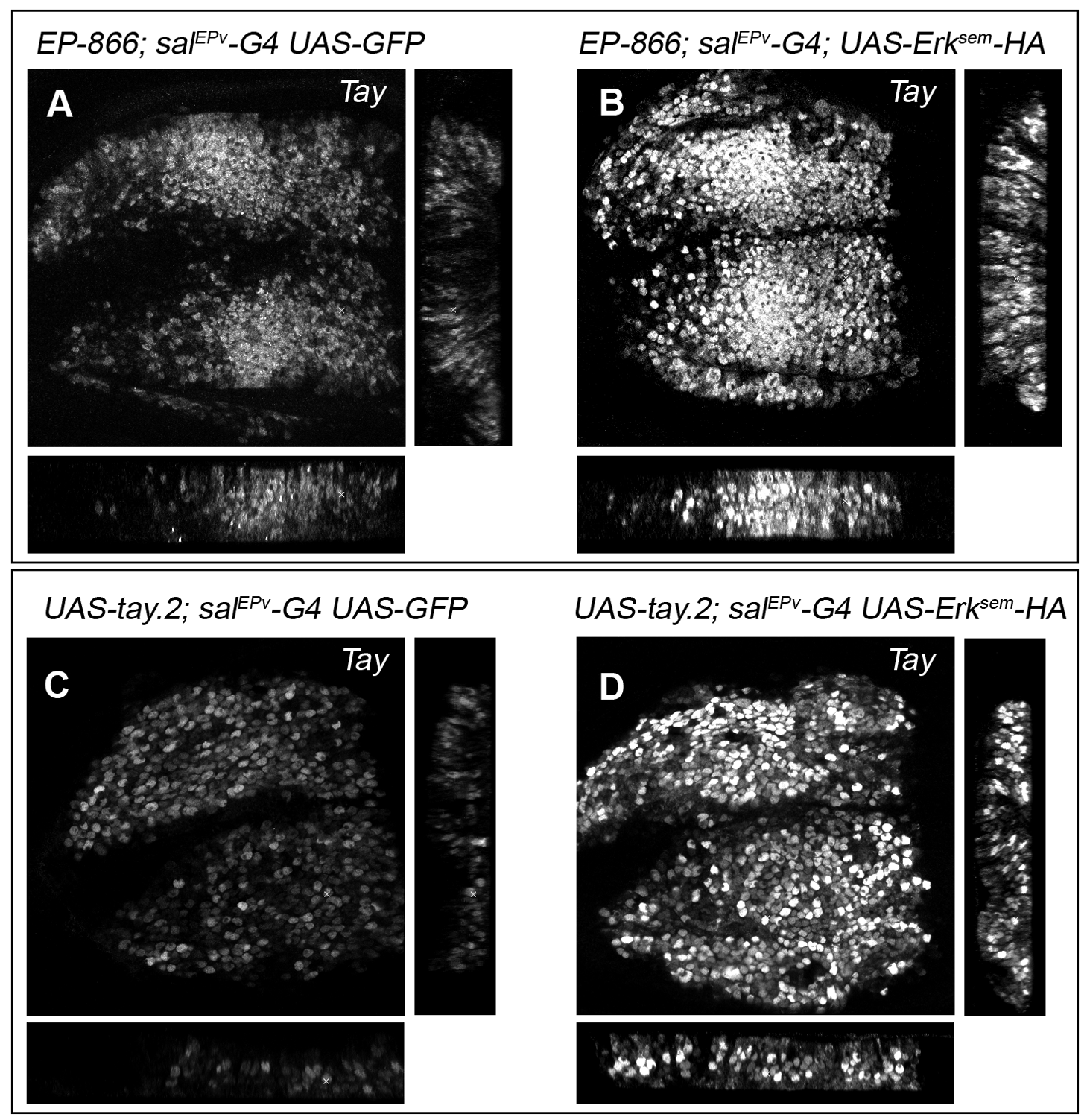

Supplement: Figure S7 — Changes in Tay and Tay.2 accumulation in response to Erksem. (A–B) Expression of Tay in control EP-866; salEPv-Gal4 UAS-GFP/+ (A) and in wing discs over-expressing Erksem (EP-866; salEPv-Gal4 UAS-ERKsem /+; B). (C–D) Expression of Tay.2 in control UAS-tay.2-Flag; salEPv-Gal4 UAS-GFP/+ (C) and in wing discs over-expressing also Erksem (UAS-tay.2-Flag; salEPv-Gal4 UAS-Erksem /+; D). Wing discs were fixed, stained and visualised using the same conditions. Note the higher levels of Tay and Tay.2 accumulation in the presence of Erk sem. (TIF) [file pgen.1003982.s007.tif]
